# A Paradox of School Social Organization: Positive School Climate, Friendship Network Density, and Adolescent Violence

**DOI:** 10.1007/s10964-024-02034-2

**Published:** 2024-06-24

**Authors:** Nicolo P. Pinchak

**Affiliations:** 1 Centre for Social Investigation, Nuffield College, University of Oxford, Oxford, UK; 2 Leverhulme Centre for Demographic Science, University of Oxford, Oxford, UK

**Keywords:** Networks, School climate, School effects, Social capital, Social disorganization theory, Violence

## Abstract

Schools are often encouraged to foster a positive climate to reduce adolescent violence, but evidence on the effectiveness of this approach varies significantly. This study investigates the roots of this variation by testing alternative hypotheses about how positive school-level climate and school-level student friendship network density interact to shape adolescent violence perpetration. Research on informal social control and network closure suggests that the violence-reducing association of positive school climate will be enhanced among schools where students are more densely tied through their friendships. Research on youth conflict and subversion of control suggests the opposite. These hypotheses are tested with data from Waves I-II of the National Longitudinal Study of Adolescent to Adult Health (n = 11,771; 49% Female; Age mean = 15.04, SD = 1.60). Consistent with the conflict/subversion hypothesis, analyses indicate that the inverse association between positive school climate and adolescent violence is only evident among schools with a very low density of friendship ties. Strikingly, however, there is evidence that a more positive school climate is associated with increases in violence among youth attending schools with a high density of friendship ties. These findings suggest that efforts to reduce violence by fostering cohesion among youth in their schools and other social contexts can be undermined by youth network processes.

## Introduction

Decades of research highlight schools as being among the most salient and influential environments in the lives of adolescents (Witherspoon et al., 2023). A significant thrust of this literature focuses on how schools can be organized to reduce adolescent violence perpetration, which is detrimental to the well-being of perpetrators, victims, and society more generally (Centers for Disease Control and Prevention [CDC], 2015). In particular, a large body of research calls attention to the importance of schools having a *positive climate* in efforts to reduce youth violence (Payne & Gottfredson, 2018) and improve other youth outcomes (Wang & Degol, 2016). Nevertheless, while there is notable evidence that positive school climate is associated with reduced violence, there is also variability across school samples (Reaves et al., 2018) sometimes even yielding adverse effects on youth bullying (Konishi et al., 2017) and violence perpetration (DiPietro et al., 2015). This begs the question of under what conditions the violence-reducing association of positive school climate is most evident. To understand the roots of the variation, this study develops and tests alternative hypotheses about how the *density of student friendship ties* in a school interacts with positive school-level climate to shape adolescent violence perpetration. These tests are conducted with extensive data on adolescents’ behaviors, friendship networks, and positive school climate from Waves I-II of the National Longitudinal Study of Adolescent to Adult Health (Add Health).

### Positive School Climate and Adolescent Violence

Beyond the home, schools are the foremost institution tasked with equipping youth with prosocial models of behavior (Arum, 2000) and are the most routinely inhabited context for the vast majority of youth (Pinchak et al., 2022). Youth with stronger bonds to their schools, teachers, and fellow students tend to be remarkably less violent than similar youth reporting weaker school bonds (Sampson & Laub, 1993). Schools nevertheless vary substantially in the content of their norms, patterns of interpersonal interaction, and capacity to discourage violence, with influences beyond those of individual-level experiences. In this respect, numerous dimensions of the school environment have been found to be consequential for understanding and reducing adolescent problem behavior (Bradshaw et al., 2021).

One especially influential perspective relevant for understanding consequences of school environments is social disorganization theory. Originally conceived to study neighborhood rates of juvenile delinquency and crime (Sampson, 2012), social disorganization theory has been widely applied to study connections between schools and adolescent problem behavior (Espelage & Hong, 2019). Social disorganization perspectives suggest that problem behavior can be reduced when members of a community have a greater capacity to trust and help one another in the face of problems, and that this capacity is hindered when communities are more structurally disadvantaged. For example, in a study testing social disorganization theory among youth in 95 schools, it was found that schools with higher student-teacher ratios and rates of poverty and suspension experienced more bullying (Bradshaw et al., 2009), and another study found that effects of school-level indicators of disorganization on adolescent violence operate indirectly through effects on students’ individual-level school experiences (Lindstrom Johnson et al., 2017).

Optimistically, however, social disorganization perspectives further suggest that the violent consequences of disadvantage can be overcome when community members foster social cohesion and strong norms about informal control of antisocial behavior. This optimism about the power of community collaboration has underpinned a plethora of school initiatives aiming to resolve problems by fostering a more *positive school climate*. The concept of positive school climate is widely recognized as abstract and multifaceted, but nearly all efforts to measure it aim to capture the extent of “supportive relationships among school community members, a common set of [prosocial] goals and norms, and a sense of collaboration and involvement” in a school (Payne & Gottfredson, 2018, p. 12), most often assessed as the aggregate of students’ perceptions of their school environments and experiences (Lindstrom Johnson et al., 2017). In schools with such positive climates, students and staff are thought to have stronger bonds to one another and be better equipped to sanction violent behavior, which in turn reinforces more prosocial strategies to achieve goals (Payne & Gottfredson, 2018). For example, in a study noting that “school climate … parallels research on social disorganization and collective efficacy,” it was found that, net of school-level poverty and individual-level school experiences, students experience less victimization when their school has more “social cohesion,” measured as the positivity of interactions among students and teachers (Zaykowski & Gunter, 2012, p. 447). Another study found that adolescents perpetrate less violence over time when attending schools characterized by more positive student-perceived connectedness, safety, and teacher fairness, even when controlling average school class sizes and urbanicity (Brookmeyer et al., 2006). A national study of schools found that higher student-teacher ratios increases students’ risk of being victimized at school, and that this association is partially explained by the extent of prosocial values among students in a school (Gottfredson & DiPietro, 2011). Likewise, a study of Kentucky adolescents found that those attending higher poverty schools are more likely to bring a weapon to school (Wilcox & Clayton, 2001). This association was furthermore mediated by school-level indicators of social capital (e.g., mean school attachment), which were argued to operate by bolstering “effective networks of control among students, parents, teachers, and administrators” (Wilcox & Clayton, 2001, p. 517).

The mechanisms linking positive school-level climate to individual-level violence remain the subject of investigation, but these are thought to be aligned with those of social control (Hirschi, 1969), ecological systems (Bronfenbrenner, 1979), and social learning (Moffitt, 1993) perspectives on violence. All these perspectives highlight the importance of youth attending schools that are cooperative and welcoming rather than conflicted (Wang & Degol, 2016), and where youth are more exposed to “conventional” and “non-delinquent” models of behavior (Thornberry & Krohn, 2017), which are evidently most prevalent in schools with positive climates. For example, in a recent review of the school climate literature, it was suggested that “strategies that increase social bonds between students and others in their schools will reduce misbehavior by increasing informal controls” (Payne & Gottfredson, 2018, p. 8). Consistent with this, one study found that youth in schools where teachers report a greater capacity to regulate behavior are less likely to be suspended or come into contact with the criminal justice system (Kirk, 2009). Some research additionally finds that students’ individual-level school experiences can mediate associations of positive school-level climate on violence perpetration (Loukas et al., 2006) and students’ willingness to report others’ misbehavior (Slocum et al., 2017).

In light of this evidence, and provided that school climate features are much more malleable than are school structural factors, school climate improvement efforts are increasingly recognized as practical strategies to enhance youth outcomes. Positive school climate has even become the subject of policy initiatives aiming to reduce youth violence and improve school safety (Bradshaw et al., 2021). For instance, the U.S. Every Student Succeeds Act (2015) acknowledges the importance of positive school climate and requires that data related to climate be included in state-issued school report cards (U.S. Department of Education, 2016). School climate initiates have additionally been suggested as potentially important for reducing school discipline problems (Valdebenito et al., 2023) and preventing school shootings (Kupchik et al., 2015). Nevertheless, some studies find inconsequential or even adverse associations between positive school climate and student problem behavior (Reaves et al., 2018). For example, one study found that youth report less bullying in schools where students are more collaborative and where teachers are seen as more fair, but also that bullying is more common in schools where students feel more cared for by adults (Konishi et al., 2017). Another study found that immigrant youth attending schools with a more positive “social climate” are more involved in violence (DiPietro et al., 2015). The question of why positive school climate is not reliably associated with reduced violence is thus a key question facing the field. Numerous answers have been proposed, such as differences in the measurement of climate (Payne & Gottfredson, 2018) and the implementation fidelity of school climate interventions (Payne, 2024). Seldom considered, however, is how other processes varying between schools may enhance or limit the association between positive school climate and adolescent violence.

### School Friendship Network Closure and Positive School Climate

In addition to climate, network processes are also central to school social organization (McFarland et al., 2014) and have long been thought to be important for understanding the conditions under which school factors shape adolescent behavior. Especially relevant is the density of students’ friendship ties in a school, classroom, or friendship group, albeit with conflicting accounts of how this density shapes violence. One set of expectations arises from Coleman’s formative studies of schools, which found that schools vary in the content and range of activities that youth reward and sanction (e.g., sports vs. academics), and that these norms about behavior form and affect students when they are densely tied to one another through their friendships (Coleman, 1961), or when network “closure” among students is high (Coleman, 1988).^1^ Recent research supports this hypothesis, finding that when individuals are embedded in more dense networks, they are more likely to sanction norm violators and be rewarded for doing so (Jan Piskorski & Gorbatâi, 2017). In addition to this sanctioning, Coleman argued that dense ties within a community builds “the trustworthiness … that allows the proliferation of obligations and expectations” (Coleman, 1988, p. S107). Indeed, students tend to be more densely tied to one another through their friendships in schools with more positive climates (McFarland et al., 2014), and have improved “trust-related outcomes” such as less conflict with peers (Allcott et al., 2007, p. 85).

**Fig. 1:**
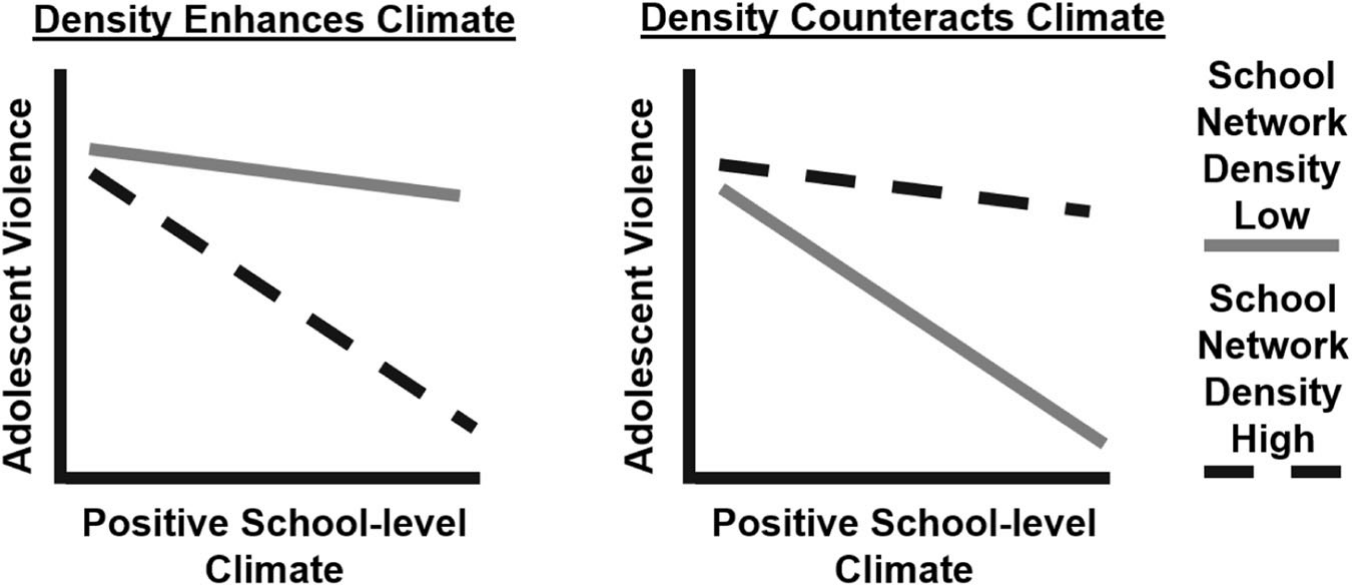
Hypothetical Illustrations of the Alternative Hypotheses

Nevertheless, there is growing acknowledgment of the neutrality of dense friendship ties within a community—as well as other dimensions of social capital—in shaping individuals’ behavior and well-being (Portes, 2014). For example, one study found that youth with more dense friendship networks tend to be less delinquent, but also that the effect of friends’ delinquency on an individuals’ delinquency is most evident for youth in highly dense friendship networks (Haynie, 2001). Another study similarly found that the density of individuals’ friendship networks is positively associated with mental well-being, but only when the network is self-affirming (e.g., where individuals can ‘be themselves’) (Walker, 2015). Likewise, the systemic formulation social disorganization theory, which draws on Coleman’s work, suggests that more dense ties among members of a community results in a greater capacity to regulate local problems, but only among communities with a strong informal control norms (Bursik, 1999). Aligning with these insights, more dense ties among students may reinforce the “informal control” processes thought to arise in schools with positive climates, such as through peer sanctioning (Payne & Gottfredson, 2018, p. 8). For instance, one study found that a school climate intervention teaching youth to sanction bullying was most successful in schools where more “social referents”—individuals with a particularly high influence on community perceptions of norms for behavior—were involved in the implementation (Paluck et al., 2016, p. 567). These is thus reason to expect that the violence-reducing association of positive school climate is empowered among schools with a high density of student friendship ties. This expectation is illustrated in the first panel of Fig. 1.

In sharp contrast however, there is reason to expect that highly dense friendship ties among youth could impede the violence-reducing benefits of a positive school climate. This possibility aligns with growing evidence on the “dark side” of social capital (Villalonga-Olives & Kawachi, 2017), which suggests that dense friendship networks can have strikingly adverse effects on individuals’ behaviors. With respect to adolescents and schools, this is perhaps most evident in the literature on bullying (Donoghue, 2022), underlining the potential for close friendships to be a major source of both support and victimization (Banny et al., 2011). For instance, adolescents can often effectively bully each other to climb the status hierarchy of their school (Faris, 2012), and they are most likely to target closely tied friends in doing so (Faris et al., 2020). Indeed, in a study of aggression in 14 schools it was observed that “aggression between friends and friends-of-friends holds great potential to entangle the many students who are adjacent to these antagonisms: in all but the five largest (and least dense) schools, between 10 and 26% of students are within two friendship links of such a conflict” (Faris et al., 2020, p. 675–676). Research moreover finds that dense friendship ties among youth are associated with reduced self-efficacy, but also greater symptoms of distress (Walker, 2015). Similarly, anti-bullying programs involving close contact between students—such as peer mentoring and mediation—have been found to sometimes inadvertently *increase* conflicts and bullying (Ttofi & Farrington, 2011).

More dense ties among individuals in a community can also evidently undermine informal controls. For instance, in a review of the social capital literature, it was noted that “[t]he capacity of authorities to enforce rules (social control) can … be jeopardized by the existence of tight networks whose function is precisely to facilitate violation of those rules for private benefit” (Portes, 1998, p. 15). Consistent with this, a study of a high-profile rape perpetrated by high school athletes against their classmate found that the offenders effectively evaded sanctions because they were protected by their highly dense school friendship network (Lefkowitz, 1997). Indeed, a particularly remarkable aspect of this case was the complicity of other students, with one researcher finding: “The newspapers were saying that accounts of what had happened had circulated among the students in the high school for almost three months before the arrests, but that the kids had kept it to themselves” (Lefkowitz, 1997, p. 4). In investigating how the adults were so unaware, it was furthermore found that: “The jocks … were sustained by an impenetrable, subterranean youth culture whose members were bound by a code of secrecy” (Lefkowitz, 1997, p. 492). Research on school shootings has similarly found that dense friendship ties among students in a school can give rise to a close-knit adolescent code, which can disincentivize students from reporting even highly credible threats to adults (Newman et al., 2008). These dynamics have also been observed in the unfolding of everyday interactions within classrooms, with research finding that a high classroom-level density of student friendship ties increases the likelihood that students will disrupt their teacher (McFarland, 2001). These findings suggest that students in schools with a high density of friendship ties are especially well-positioned to subvert the informal controls thought to result from a positive school climate.

Beyond youth and schools, adverse consequences of dense ties within a community are also evident in the broader literature on social capital and disorganization. For example, one study found that gangs contributing to the well-being of their community were nearly immune to informal social control efforts (Pattillo, 1998). This immunity was driven in part by gang involvement among local youth, whom many residents wanted to protect from the punitive sanctions of law enforcement, and which these youth exploited to subvert control. Building on this insight, another study found that the crime-reducing benefits of social control norms are least evident in neighborhoods with more positive network ties among residents (Browning et al., 2004). The authors concluded that dense ties among members of a community can thus simultaneously promote social control and “enhance the social capital of offenders” (Browning, 2009, p. 1572). In sum, these studies of community, school, and youth network processes suggest that a high density of student friendship ties in a school can hinder the informal control mechanisms of positive school climate, such that the inverse association between positive school climate and violence is most evident among schools where students are relatively sparsely tied to one another through their friendships. This expectation is illustrated in the second panel of Fig. 1.

## Current Study

The literature motivates two conflicting hypotheses about how the association between positive school climate and adolescent violence perpetration depends on the density of student friendship ties in a school. On the one hand, youth attending schools characterized by more dense student friendship ties are better able to enforce norms about one another’s behavior, which could enhance the violence-reducing association of a positive school climate. However, more dense friendship ties can also lead to more conflicts among youth and be used to subvert informal controls, which could counteract positive school climate such that its inverse association with violence is most evident among schools characterized by sparse friendship ties. These hypotheses have potentially significant implications for research on schools and adolescents, such as by yielding divergent expectations about the conditions under which positive school climate interventions are optimized to improve youth behavior, and about how network processes can empower or disrupt informal social control efforts seeking to reduce violence. Using data from Add Health, this study tests these hypotheses by assessing the interaction between positive school-level climate and school-level density of student friendship ties in predicting adolescent violence perpetration.

## Methods

### Data and Sample

Data are from Waves I and II of Add Health, a nationally representative school-based study of adolescents in the United States (Harris et al., 2009). The sampling frame included 80 high schools and additional feeder middle schools stratified by region, urbanicity, sector, and size. The Wave I longitudinal sample (n = 20,745) consists of adolescents in grades 7–12 in 1994–95. Wave II (n = 14,738) was collected one year later in 1996 and consists of Wave I respondents still enrolled in school (e.g., excluding Wave I high school seniors). School-level data were collected from school administrators, by linking data from the National Center for Education Statistics via the Adolescent Health and Academic Achievement study, and from schoolwide student surveys (n = 90,118) issued from September 1994–April 1995 prior to the Wave I in-home interview. These in-school surveys make Add Health data extraordinarily well-suited to test the hypotheses, with students being asked about their perceptions of school climate and their friendship ties.

### Measures

#### Violence

Adolescent Violence at Waves I and II is estimated using responses to seven survey questions about perpetration of the following within the past twelve months: been involved in a serious fight, hurt someone badly enough to need medical attention, participated in a group-on-group fight, used or threatened to use a weapon to get something from someone, pulled a weapon on someone, shot or stabbed someone, or, in the last 30 days, carried a weapon (such as a gun, knife, or club) to school. Each of these were recoded as binary indicators (1 = committed the act, 0 = did not commit the act). Consistent with conventions in the adolescent violence literature (e.g., Sharkey & Sampson, 2010), items were then entered as dependent variables into a multilevel Rasch model where violence reports are clustered within respondents (Raudenbush et al., 2003). Like Rasch models estimated in the context of ability testing, Rasch models applied to dichotomous violence indicators of varying degrees of severity—or rarity, e.g., fights are more common than weapon use—can be understood as estimating a respondent’s latent propensity for violence, and allows for missingness on responses to any given violence item. The respondent-level empirical Bayes estimate from this model was recovered to yield a respondent violence score, and this procedure was carried out separately by wave based on all responses given at each time point.^2^ The distributions of Waves I and II violence perpetration items in the full and analytic samples are displayed in Appendix Table 1. The final measures are z-score standardized for ease of interpretation (i.e., grand mean centered at 0, SD = 1). Additionally, the distribution of the Wave II violence scores in the analytic sample is presented in Appendix Fig. 1.

#### School-level positive school climate

Positive school climate is measured using student self-reported data from the In-school survey. Questions were selected because of their alignment with salient school climate theory and literature emphasizing students’ experiences and perceptions (Kohl et al., 2013) of school belonging (Payne & Gottfredson, 2018), positive interpersonal relationships among students and teachers (Zaykowski & Gunter, 2012), and clarity and consistency of school rule enforcement (Gerlinger & Wo, 2016). Selected items include “How often since the school year started did you have trouble:” (1) “getting along with teachers,” (2) “getting along with other students,” and “how strongly do you agree or disagree with the following statement:” (3) “I feel close to people at this school” (4) “I feel like I am part of this school,” (5) “I am happy to be at this school,” and (6) “the teachers at this school treat students fairly.”^3^ All item response options range from 0 to 4 and were recoded such that higher response categories represent more positive school experiences. Responses were entered as dependent variables into a multilevel linear 1-parameter (for item ‘difficulty’) model with reports clustered in respondents and respondents clustered in schools, and the derived school-level empirical Bayes estimate is used to measure school-level positive climate (Raudenbush & Bryk, 2002). The school-level reliability for this measure, calculated using Raudenbush and Sampson’s (1999) equation for assessing multilevel reliability, is 0.66.^4^ Additionally, when each survey measure is modeled separately using two-level models, the Cronbach’s alpha coefficient for the resulting school-level measures is 0.75.

While there is strong theoretical and empirical precedent for focusing on consequences of a single, holistic measure of positive school climate (e.g., Brookmeyer et al., 2006), some research suggests that student-perceived positive school-level climate is multidimensional (Crosnoe et al., 2004). This possibility motivates a principal component analysis of the six school-level climate measures, which was conducted in Stata 18 with varimax rotation. This analysis yields evidence for use of two school-level climate component measures. Together these two components account for 88% of the total variance among the six school-level climate measures, and 99% of the variance in the holistic school-level measure.

##### School community attachment

The first component, titled “school community attachment,” has an eigenvalue of 3.31 and corresponds to the four survey questions about students’ perceptions of fairness, closeness, happiness, and inclusion at school.

##### Low interpersonal trouble

The second component, titled “low interpersonal trouble,” has an eigenvalue of 1.95 and corresponds to the two survey questions about students’ extent of trouble getting along with teachers and other students.

#### School-level friendship network density

School friendship network density is based on data from the in-school questionnaire, which asked respondents to nominate up to five female and male friends from a roster of all students enrolled in either their current school or the sampled “sister” school (e.g., a high school and feeder middle school pair). From these data, school-level measures of student network density can be created. Because of a heavily skewed distribution of student population sizes across schools (i.e., a maximum of 10 nominated friends is meaningfully different depending on school size), a measure of “relative density” is used in the present analyses, and outlined by the authors of the Add Health Network Variables Codebook (Carolina Population Center, University of North Carolina, 2001). These authors are also followed in assessing consequences of relative density only among youth in schools where at least 50% of the students completed the In-school survey.^5^

First, school-level network density is defined as the number of ties in the total school network divided by the number of possible ties in the total school network:

$${\rm{Density}}=\frac{\sum {\rm{X}}}{{\rm{g}}* ({\rm{g}}-1)}$$

Where ${\rm{X}}$ is the total school network and ${\rm{g}}$ is the number of nodes in ${\rm{X}}$. Relative density is then defined as “observed density divided by maximum possible density given out-degree = 10” (Carolina Population Center, University of North Carolina, 2001, p. 20), with the school-level equation given as:

$${\rm{Relative\; Density}}=\frac{{\rm{Density}}}{[(10* {\rm{g}})/({\rm{g}}* \left({\rm{g}}-1\right))]}$$

#### School-level socioeconomic disadvantage

School socioeconomic disadvantage is an index combining the school-level proportion of students receiving free or reduced-price lunch (FRPL), the proportion of students whose parents reported receiving “public assistance, such as welfare,” and the proportion of students whose parents have not completed a high school education (Pinchak & Swisher, 2022). These items were z-score standardized and averaged across schools with at least one non-missing indicator (alpha among schools = 0.88), and the final measure is z-score standardized.

#### School-level student population

School-level student population is based primarily on data from the NCES, and on the Wave I In-school survey count of students when NCES data are missing.

#### School-level pupil-teacher ratio

Pupil-teacher ratio is based primarily on NCES reports, and on school administrator reports of average class size when NCES data are missing.

#### School-level suspension rate

The school suspension rate is the proportion of students in the school who report having ever received an out-of-school suspension.

#### School-level %Black

The percent of students in a school who are Black is based primarily on NCES data and on in-school survey reports when NCES measures are missing.

#### School-level %Hispanic

The percent of students in a school who are Hispanic is based primarily on NCES data and on in-school survey reports when NCES measures are missing.

#### School-level private

Private school is a binary indicator for whether the school is private vs. public.

#### School-level County density

The population density of the county in which the school is located is used to control urbanicity.

#### Individual-level school climate measures

Four individual-level school climate measures were created based on the following Wave I In-home survey reports, all of which have response options range from 0 to 4 and were recoded such that higher response categories correspond to more positive school experiences. When asked of respondents during the summer, students were asked these questions about school experiences “last year.”

##### Trouble with teachers

Trouble with teachers is based on responses to “How often since the school year started did you have trouble getting along with teachers.”

##### Trouble with students

Trouble with students is based on responses to “How often since the school year started did you have trouble getting along with other students.”

##### School attachment

School attachment is the average of the following three questions asking “how much do you agree or disagree with the following statements:” “I feel close to people at this school,” “I feel like I am part of this school,” and “I am happy to be at this school” (alpha = 0.77).

##### Teacher fairness

Teacher fairness is based on responses to “How much do you agree or disagree with the following statements: The teachers at your school treat students fairly.”

#### Individual-level GPA

GPA (grade point average) is based on self-reported grades in science, math, social studies, and English over the past (Wave I) year (alpha = 0.74).

#### Individual-level suspended

Having ever received an out-of-school suspension is self-reported and controlled given evidence of complex, bidirectional relationships between school punishment (Duxbury & Haynie, 2020), perceptions of school climate (Del Toro & Wang, 2022), and adolescent problem behavior (Jacobsen, 2020).

#### Individual-level personal network size

Based on data from the in-school interview, personal network size is the total number of a respondent’s received and sent friendship nominations plus themself.

#### Individual-level no network data

Because a substantial proportion of longitudinal respondents did not partake in the in-school survey (e.g., were sampled subsequent to the in-school survey), a binary variable for no network data was created, for whom the personal network size equals 1. For more information on how the in-school and in-home samples were determined, see citations: Carolina Population Center, University of North Carolina (2001) and Harris (2013).

#### Individual-level parent relationship quality

Parent relationship quality is a z-score standardized measure combining adolescents’ responses to 16 Likert scale survey questions about perceptions of love, closeness, warmth, communication, and educational expectations from the mother, father, and “family” as a whole (alpha = 0.87; see Appendix Table 2).

#### Individual-level impulsivity

Impulsivity is a z-score standardized measure combining self-reported responses to four Likert scale questions about approaches to problems (alpha = 0.74; see Appendix Table 2).

#### Individual-level neighborhood monitoring

Neighborhood monitoring is based on three binary survey questions asking: “do you usually feel safe in your neighborhood,” “people in this neighborhood look out for each other,” and “you know most of the people in your neighborhood.” All responses are coded such that an affirmative response corresponds to more monitoring, and available responses are averaged together (alpha = 0.45).

#### Individual-level neighborhood disadvantage

Neighborhood disadvantage combines census tract measures of proportion of children in a family below the poverty line, the proportion of adult residents living below the poverty line, the proportion of female headed households with children, and the unemployment rate. These items were averaged together (alpha = 0.88), and this measure was then z-score standardized.

#### Individual-level neighborhood instability

Neighborhood residential instability is the mean of two census tract measures including the proportion of residents who do not own their place of residence and the proportion of residents who moved into the tract during the last 5-years (correlation = 0.59).

#### Individual-level family socioeconomic status

Family socioeconomic status is measured using an approach where the present mother’s and father’s highest levels of education and occupational attainment were used to create two 5-category variables (see Bearman et al., 2004). These were then added together, and the final measure is z-score standardized.

#### Individual-level family structure

Lived with two biological parents is a binary indicator of whether the adolescent resided with both their biological parents vs. some other family living situation at Wave I.

#### Individual-level race-ethnicity

Mutually exclusive race-ethnicity categories are based on self-reported data and include non-Hispanic white, non-Hispanic Black, any Hispanic, non-Hispanic Asian, and some other race.

#### Individual-level sex

Biological sex is self-reported.

#### Individual-level age

Age is self-reported and calculated in years.

### Analytic Strategy and Sample

In order to make necessary adjustments for the clustering and unequal probability of selection among Add Health respondents (Chen & Harris, 2020), violence at Wave II is modeled using linear (Sharkey & Sampson, 2010) single-level “population average” models weighted using the “svyset” command in Stata 18, specifying schools as the primary sampling unit in which students are clustered, regions of the U.S. as the strata cluster variable, and estimating Taylor-linearized (i.e., “robust”) standard errors (StataCorp, 2023b).^6^ These weights are grand sampling weights, which account for the multistage sampling design and attrition using a single weight variable. This approach is deferred to particularly because “[f]ailure to account for the sampling design usually leads to underestimating standard errors and false-positive statistical test results” (Chen & Harris, 2020, p. 2). Interaction terms are assessed by estimating average marginal effects of the interacted variables at levels of one another—e.g., the expected unit change in violence associated with a one-unit increase in positive school climate among youth attending schools at the 10^th^ vs. 90^th^ percentile of relative network density (Mize, 2019; Mize et al., 2019).

The analytic sample is restricted to Wave II respondents (n = 14,738) with nonmissing sampling weights and poststratification region variables (n = 13,568) and who, at Wave I, attended an Add Health school that is not missing on school-level relative network density (n = 11,771; school n = 113). Among this sample, the average number of respondents clustered within a school is 104.2, the minimum is 18, and the maximum is 1,105. 5% of the sample is missing on at least one independent variable. These respondents are retained by using multiple imputation by chained equation procedures with 20 imputed datasets (von Hippel, 2020). This procedure generates values for missing variables drawn at random from a posterior predictive distribution conditioned on the observed values of the missing variables and other variables in the analysis (in this case, all the measures discussed previously) (White et al., 2011). Analyses are then conducted on the imputed datasets and combined by adjusting coefficients and standard errors for the variability between imputations (Rubin, 1987; StataCorp, 2023a).

The analyses proceed in three steps. First, in alignment with the vast majority of studies on positive school climate and adolescent violence, the interaction between school-level relative network density and positive climate is assessed in a cross-sectional analysis of Wave II violence. This is followed by a more rigorous longitudinal analysis of change in adolescent violence by controlling Wave I violence. Finally, the longitudinal analyses are replicated when controlling adolescents’ individual-level perceptions of school climate, which some studies suggest could mediate associations of positive school-level climate with violence. Finally, numerous sensitivity analyses are conducted, including analyses using the alternative positive school climate measures, tests of whether results differ by respondent sex, and whether results are robust to inclusion of interactions between positive school climate and measures of adolescents’ personal friendship network resources.

## Results

Table 1 displays descriptive statistics for all study variables. The correlation between school-level positive climate and relative network density is 0.43. A scatter plot of these measures is presented in Appendix Fig. 2, where representative values of relative network density are also reported in their original (non-standardized) metric. Although all the school- and individual-level control measures used for this analysis are standard in this literature, variance inflation factors (VIFs) were assessed among the independent variables in the analytic sample to understand the degree of multicollinearity (Thompson et al., 2017) (except for dummy-coded race categories and multiplicative terms; Allison, 2012). The VIF for positive school climate is 2.65, 2.78 for relative network density, and the average VIF is 1.77. Use of VIF threshold values to assess and diagnose multicollinearity are widely debated, but some research suggests that VIFs greater than 5 are particularly problematic for inference (Kalnins & Praitis Hill, 2023), though others suggest that VIFs greater than 2.5 for focal independent variables can be concerning (Allison, 2012). Given that VIFs for positive school climate and relative network density exceed 2.5, a sensitivity analysis is conducted including only pupil-teacher ratio and suspension rates as school-level control variables, which yields a VIF of 2.37 for positive school climate, 1.86 for relative network density, and a mean VIF of 1.42. These two school-level controls were retained for this sensitivity analysis because they most directly capture the two school processes that are most likely to confound associations between positive school climate and student behavior, including the number of adults available to regulate student behavior in a school (Arum & LaFree, 2008) and the extent of problem behavior and punitiveness in a school (Bradshaw et al., 2009).^7^

**Table 1:** Descriptive statistics for study variables

	Weighted Mean	SD	Min.	Max.
Dependent Variables				
Wave II Violence	0.03	1.00	−0.60	3.85
Wave I Violence	0.02	1.00	−0.78	3.56
School-level measures				
Positive school climate	−0.03	1.00	−3.03	3.09
PCA: Low interpersonal trouble	−0.17	1.00	−2.69	2.54
PCA: Community Attachment	0.10	1.00	−2.42	3.09
Relative network density	0.19	1.00	−2.15	3.89
School socioeconomic disadvantage	0.04	1.00	−1.63	3.46
School %Black	−0.07	1.00	−0.79	2.98
School %Hispanic	−0.23	1.00	−0.64	3.75
Pupil-teacher ratio	17.99	4.31	10	30
School suspension rate	0.29	0.14	0.03	0.68
School student population	968	750	52	3350
Private school (vs. public school)	0.06	–	0	1
County density (persons/sq. km.)	0.42	1.45	0	12.59
Individual-level measures				
Neighborhood disadvantage	0.02	1.00	−1.47	7.90
Neighborhood residential instability	−0.01	1.00	−2.17	4.35
Parent relationship quality	0.03	1.00	−5.36	1.40
Neighborhood monitoring	0.05	1.00	−2.75	0.79
Impulsivity	0.05	1.00	−1.91	4.48
Interpersonal trouble with students	0.06	1.00	−0.90	3.18
Interpersonal trouble with teachers	0.06	1.00	−0.91	3.21
School attachment	0.01	1.00	−3.23	1.44
Teacher fairness	0.00	1.00	−2.34	1.41
GPA	0.10	1.00	−2.53	1.52
Suspended	0.28	–	0	1
Personal network size	6.83	4.88	1	33
No network data	0.23	–	0	1
Age at Wave I	15.04	1.60	11	20
Female	0.49	–	0	1
Race				
White	0.66	–	0	1
Black	0.17	–	0	1
Hispanic	0.10	–	0	1
Asian	0.04	–	0	1
Other race/ethnicity	0.04	–	0	1
Family socioeconomic status	−0.07	1.00	−2.54	1.20
Lives with two biological parents	0.56	–	0	1
Sample N	11771			

Means are weighted using Add Health sampling weights. Standard deviations not shown for binary variables

**Fig. 2:**
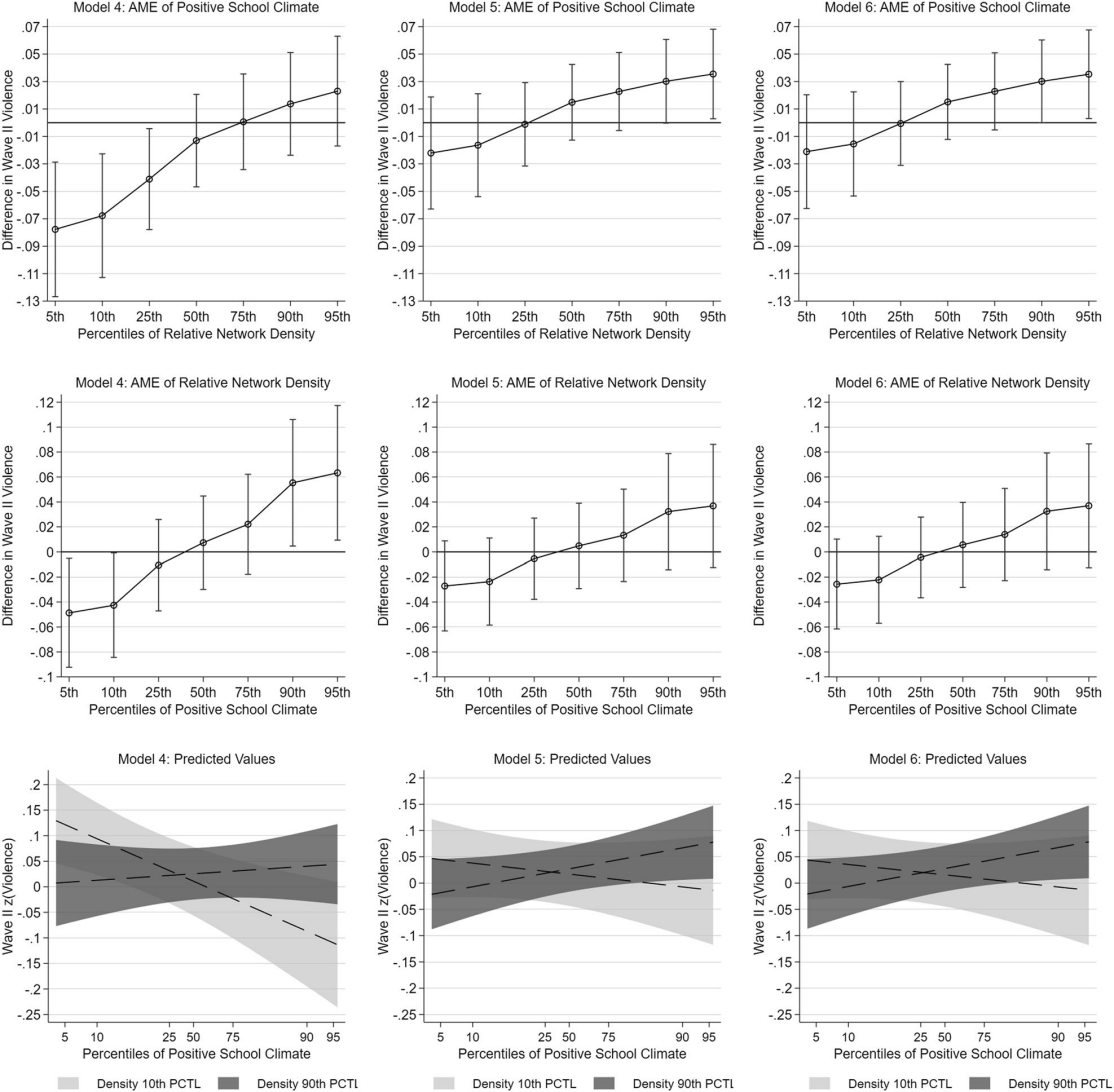
Models 4–6: Average Marginal Effects (AME) of Positive School Climate and Relative Network Density at Percentiles of One Another and Predicted Values

For efficiency, only the coefficients and standard errors for school-level climate measures and relative network density are featured, but the full tables are presented in Appendix Table 3. Table 2 presents coefficients and standard errors for school-level positive climate, relative network density, and the interaction term between these from weighted linear models for adolescent violence perpetration. Models 1–4 are cross-sectional model for Wave II violence, while Models 5–8 additionally control Wave I violence. Model 1 indicates that positive school climate does not have a statistically significant association with Wave II violence perpetration net of the control variables. Model 2 removes positive school climate and adds school-level relative network density, which also does not have a statistically significant association with Wave II violence net of controls. When both measures are included in Model 3, there is again minimal evidence that either school-level measure is associated with the outcome. Central to the hypotheses, Model 4 adds the interaction term between positive school climate and relative network density, which is positive (b = 0.033) and statistically significant (p < 0.001). To evaluate this interaction, the left-side column of Fig. 2 display average marginal effects and 95% confidence intervals for positive school climate and relative network density at the 5th, 10th, 25th, 50th, 75th, 90th, and 95th percentiles of each other. At the bottom of this column are predicted values from this model with 95% confidence intervals for the association of positive school climate among respondents at the 10th and 90th percentiles of network density when all other variables are held at their means. The top left figure indicates that the violence-reducing association of positive school climate is most evident among schools low in relative network density (e.g., below the 50th percentile). For example, among respondents at the 10th percentile of relative network density, a one standard deviation increase in positive school climate is associated with an expected −0.067 of a standard deviation decrease (p < 0.01) in violence perpetration. In this model, there is also evidence that relative network density has a statistically significant negative association with violence perpetration when positive school climate is low (e.g., at the 10th percentile), although the association is positive for youth attending with schools with very positive climates.

**Table 2:** Weighted linear models for adolescent violence

	1	2	3	4	5	6	7	8
School-level Social Processes								
Positive school climate	−0.019 (0.019)		−0.018 (0.018)	−0.026 (0.017)	0.008 (0.014)	0.008 (0.014)		
Relative network density		−0.013 (0.021)	−0.012 (0.020)	0.007 (0.019)	0.005 (0.017)	0.006 (0.017)	0.008 (0.016)	0.001 (0.019)
Positive school climate * Relative network density				0.033*** (0.009)	0.019* (0.008)	0.018* (0.008)		
PCA School-level Climate Components								
Low Interpersonal Trouble							−0.010 (0.017)	
Low Interpersonal Trouble * Network density							0.023* (0.010)	
School Attachment								0.016 (0.015)
School Attachment * Network density								0.010 (0.011)
Wave I Violence controlled					✓	✓	✓	✓
Individual-level school climate controls						✓	✓	✓
(Intercept)	0.671 (0.889)	0.662 (0.890)	0.662 (0.887)	0.714 (0.885)	0.575 (0.763)	0.559 (0.766)	0.498 (0.785)	0.346 (0.770)
N (Respondents)	11771	11771	11771	11771	11771	11771	11771	11771
N (Schools)	113	113	113	113	113	113	113	113

***p < 0.001; *p < 0.05; +*p* < 0.1

Coefficients with errors in parentheses. All continuous independent variables are z-score standardized except for county population density, pupil-teacher ratio, school suspension rate, personal network size, age, and school student population. Models are weighted according to Add Health Guidelines using the Stata “svyset” command. See appendix for the full tables

Model 5 additionally controls Wave I violence to assess change in violence between Waves I and II. The interaction term between school climate and relative network density is again positive and statistically significant (b = 0.019, p < 0.05), and the average marginal effects and predicted values from this model are presented in the center column of Fig. 2. While the association of relative network density is now statistically nonsignificant across the distribution of school climate, the association of school climate with Wave II violence is now only statistically significant and *positive* at very high levels of relative network density. For example, among respondents at the 95th percentile of relative network density, a standard deviation increase in positive school climate is associated with an expected 0.036 standard deviation increase (p = 0.033) in Wave II violence net of the control variables (p = 0.053 at the 90th percentile of relative network density). Model 6 adds the control variables for individual-level school climate perception measures of interpersonal trouble with students, interpersonal trouble with teachers, school attachment, and teacher fairness. However, here there is little evidence of change in the interaction term between positive school climate and relative network density (b = 0.018, p < 0.05). Average marginal effects and predictions from the model are presented in the third column of Fig. 2, again yielding evidence of a positive association between positive school climate and violence among respondents of schools with high levels of relative network density. For example, among respondents at the 95^th^ percentile of relative network density in this model, a standard deviation increase in positive school climate is associated with an expected 0.035 standard deviation increase (p = 0.032) in Wave II violence net of the control variables (p = 0.050 at the 90^th^ percentile of relative network density).

**Fig. 3:**
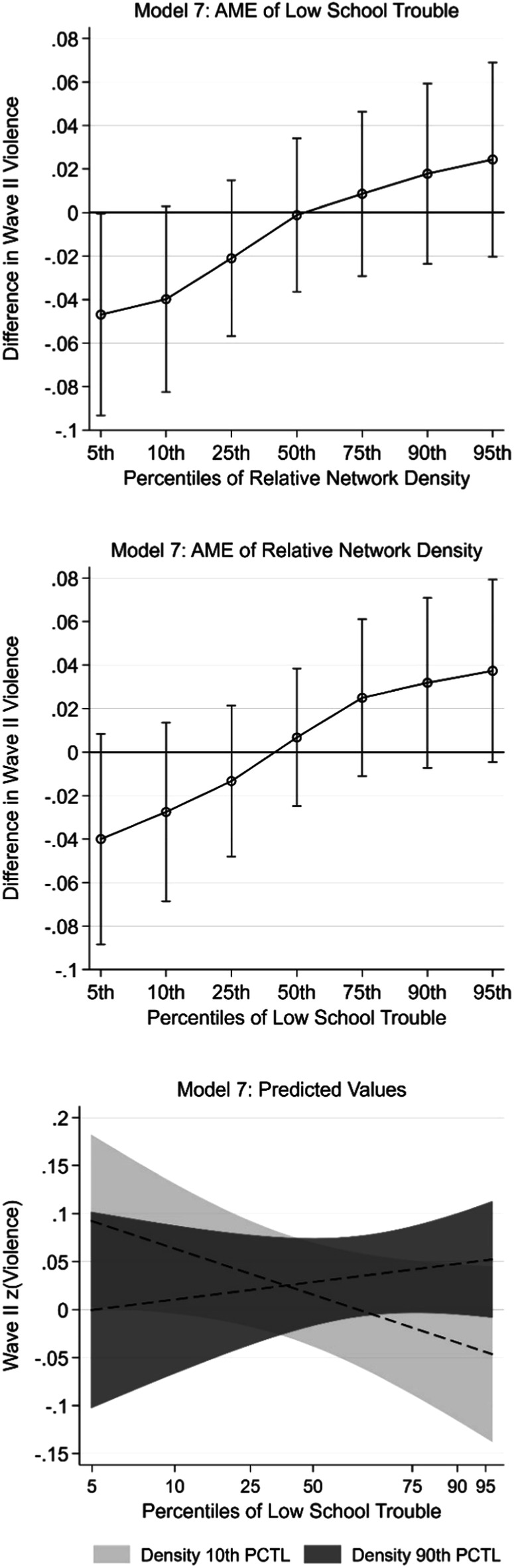
Model 7: Average Marginal Effects (AME) of Low Interpersonal Trouble and Relative Network Density at Percentiles of One Another and Predicted Values

In light of the previously discussed principal component analyses motivating two separate dimensions of positive school-level climate, the final two models replicate Model 6 when considering consequences of school-level measures of “low interpersonal trouble” and “school community attachment” in place of the holistic positive school climate measure. Turning first to consequences of low interpersonal interaction, Model 7 indicates that the interaction between this measure with relative network density is positive and statistically significant (b = 0.023, p < 0.05). To assess this interaction, average marginal effects of the two interacted variables and predictions from this model are presented in Fig. 3. Here it is evident that less interpersonal trouble in a respondent’s school is associated with reduced violence perpetration at very low levels of relatively network density. For example, among youth at the 95^th^ percentile of relative network density, a one standard deviation increase in low interpersonal trouble is associated with an expected 0.047 standard deviation decrease (p < 0.05) in violence. Moreover, the magnitude of this association is nontrivial, with an effect size equivalent to that of z-score standardized GPA in this model (b = −0.047, p < 0.001). The association of low interpersonal trouble is otherwise statistically nonsignificant and, like with the holistic measure, the direction of this association turns positive at high levels of relative network density. A similar pattern is evident for relatively network density in this model, although these associations are statistically nonsignificant across the distribution of low interpersonal trouble. Turning to Model 8, the interaction term between school community attachment and relative network density is statistically nonsignificant. Average marginal effects and predictions from this model are nevertheless presented in the center panel of Appendix Figure 3, offering no evidence that either school community attachment or relatively network density have statistically significant associations with violence at levels of one another.

In sum, these analyses indicate that positive school climate is only negatively associated with violence perpetration among schools characterized by very low levels of student friendship network density, particularly so in cross-sectional analyses and when conceiving of positive school climate as low interpersonal trouble in a school. Longitudinal analyses further indicate that the holistic measure of positive school climate is associated with *increases* in adolescent violence among youth attending schools with a high density of student friendship networks. Finally, there is little evidence that controlling individual-level school climate perceptions altered this adverse association of positive school climate.

### Supplemental Analyses

Full models and corresponding figures for average marginal effects and predictions for the analyses discussed here are presented in the Appendix. First, given the potential for problems of multicollinearity among the school-level variables, Models 6–8 were replicated when only pupil-teacher ratio and the suspension rates are included as school-level control variables. Average marginal effects and predictions from the replicated Model 6 reveal a pattern nearly identical to that presented in Fig. 3 but with slightly larger confidence intervals. Average marginal effects and predictions for the replicated Models 7 and 8 similarly yield patterns nearly identical to those discussed in the main analyses, but with more evidence of a negative association between school-level low interpersonal trouble and violence among youth attending schools low in relative network density.

Second, considering the important moderating role of gender within the school climate literature (Payne, 2009), Models 6–8 were replicated when including a three-way interaction term between female*school climate measures*network density and all lower order interaction terms. Given the skewed nature of violence measures particularly among females even when derived from Rasch models, these models were also replicated when using Poisson models with a log-link (Nichols, 2010), which can appropriately be used to model non-discrete skewed dependent variables when the minimum value is set to = 0 (Marwell & Gullickson, 2013). Results from the linear model offer some, although statistically nonsignificant, evidence that the adverse association of positive school climate is evident for both males and females of high-network density schools. However, results from the Poisson model indicate that this association is specific to male adolescents. Results when measuring school-level climate as low interpersonal trouble reveal a similar pattern, but with largely nonsignificant average marginal effects of this measure and relative network density among both males and females. A similar pattern is also evident when measuring school-level climate as school community attachment, but now with more evidence of a positive association between this measure with violence only among females attending schools with a high friendship network density.

The final set of analyses considers whether results are robust to the inclusion of interactions between respondents’ personal friendship network size and positive school climate measures and whether results vary by whether the respondent participated in the friendship network survey. Models including the interaction between personal network size*school-level climate measures yield conclusions identical to those based on the featured analyses. Results from models including interactions with the binary indicator of having personal network data indicate the adverse association of positive school climate among high-network density schools is particularly evident for respondents with no personal network data. Among these same respondents, there is also statistically significant evidence of negative associations of positive school climate and low interpersonal trouble with violence among schools low in network density, and that network density is negatively associated with violence among schools lacking in positive school climate. Overall, these supplemental analyses affirm conclusions based on the presented analyses, but with moderately more evidence of a violence-reducing association of school-level low interpersonal trouble among schools with a low density of student friendship ties.

## Discussion

Youth attending schools with a more positive climate tend to perpetrate less violence. Policymakers have taken note of this, now incentivizing schools to focus on improving their climates to improve youths’ safety and academic and social-emotional skills (U.S. Department of Education, 2016). Despite the growing relevance of positive school climate, however, this literature has continued to yield highly varied results, calling attention to the need for studies that can enlighten systematic sources of variation in associations between positive school climate and adolescent violence perpetration. To this end, this study assessed one potential source of this variation. Specifically, it tested alternative hypotheses about how the association between positive school climate and adolescent violence perpetration depends on the density of friendships among students in a school (Coleman, 1961). Research on network closure and norm enforcement largely anticipates that more dense friendship ties among students in a school empower the informal social control mechanisms of positive school climate, such as by facilitating youths’ enforcement of prosocial norms about behavior. Nevertheless, there is notable evidence that more dense friendship ties among youth can exacerbate interpersonal conflicts (Faris et al., 2020) and facilitate evasion of sanctioning (Lefkowitz, 1997), which suggests that the violence-reducing association of a positive school climate is specific to schools with a low density of student friendship ties.

The analyses reveal three findings of importance to the literatures on adolescent violence, schools, and informal social control dynamics. First, and consistent only with the conflict/subversion perspective, the negative association between the holistic measure of positive school climate and violence perpetration was evident only among schools with a very low density of friendship ties, and this association was largely only evident in cross-sectional analyses. Although less rigorous than the longitudinal results, this finding is still significant, as it aligns with the largely cross-sectional evidence base of school climate initiatives and informs efforts to describe the educational ecology of adolescent violence (e.g., what kinds of schools experience more violence). Second, longitudinal analyses further indicate that the holistic measure of positive school climate is associated with *increases* in adolescent violence among youth attending schools with a high density of student friendship ties. Paradoxical to the control/closure hypothesis, this finding suggests that highly dense friendship ties among students in a school activate an adverse association of positive school climate with adolescent behavior. Moreover, there was minimal indication that individual-level school climate perceptions explained this adverse association, suggesting that its mechanisms are beyond students’ personal perceptions of school community attachment, teacher fairness, or trouble getting along with other students and teachers. Finally, longitudinal analyses measuring positive school climate specifically as low school-level rates of interpersonal conflict indicate that this measure is associated with reduced violence perpetration among schools with a very low density of student friendship ties. This finding again affirms the conflict/subversion hypothesis, but underscores that the violence-reducing role of positive school climate is only evident when it is measured as the absence of conflict in a school.

These findings have critical implications for initiatives seeking to organize schools to reduce adolescent violence. Most obviously, the present findings do not yield optimism for initiatives focused on fostering positive student-perceived school climates, particularly if these also result in more dense friendships among students. Importantly, however, not all positive school climate initiatives necessarily lead to increases in such strong relational ties. For example, school initiatives specifically targeting the spread of norms about appropriate behavior, rather than relationship building, can evidently yield large reductions in bullying (Paluck et al., 2016) and may also be a fruitful direction for schools seeking to reduce violence. This suggestion additionally aligns with insights from the broader literature on community social organization, which underlines the importance of prosocial norms and “weak ties” (Granovetter, 1973), or relationships rooted in acquaintanceship rather than intimacy, in order to reduce violence and other problems (Sampson, 2012). The present findings moreover suggest that school climate initiatives targeting violence may yield better returns when also aiming to reduce the density of student friendship ties in a school. Research finds that adolescents’ classrooms are a dominant source of their friendship ties (Frank et al., 2013), which is optimistic because classes can readily be manipulated by school administrators, and teachers have many techniques at their disposal to reduce the occurrence and consequences of dense friendships (McFarland, 2001). These are important considerations for school-based intervention programs, particularly as schools are increasingly encouraged to “create more cohesive” environments in order to optimize youth well-being (Payne & Gottfredson, 2018, p. 15).

The potential for highly dense friendship ties to counteract positive school climate in shaping violence was motivated by research on youth conflict and subversion of control, but this research also suggests a host of potential network-based mechanisms for how a positive school climate could inadvertently increase violence. For example, the adverse association of positive school climate among high-friendship network density schools may truly operate as a schoolwide processes, such as by exacerbating consequences of the positive association between intimacy and aggression (Banny et al., 2011) among friends across a school (Faris et al., 2020). Likewise, the breadth and effectiveness of students’ attempts to avoid sanctioning in schools with a high density of friendship ties may be further enhanced when combined with stronger bonds to one another. Indeed, highly cohesive, collaborative networks tend to be remarkably more capable of developing and fulfilling shared obligations among their members (Jan Piskorski & Gorbatâi, 2017).

In contrast to these suggested schoolwide processes, however, the present findings may be driven by processes directed at youth of certain positions within a school’s network hierarchy (McFarland et al., 2014). For example, schools characterized by exceptionally strong bonds and dense friendship ties may afford leeway only for popular youth to engage in problem behavior. Consistent with this, research on adolescent drug dealing (Jacques & Wright, 2015), sexual assault (Lefkowitz, 1997), and classroom disruptions (McFarland, 2001) all suggest that popular (male) adolescents are often given a pass on their antisocial behavior when embedded in highly cohesive networks, which over time could positively reinforce their use of this behavior to achieve goals (Akers & Jennings, 2019). Conversely, the present findings could be capturing consequences of processes directed at very unpopular youth, who studies suggest are highly vulnerable to victimization and sanctioning in schools characterized by highly cohesive student relationships (Donoghue, 2022), and are in turn at a particularly high risk of engaging in violence (Kreager, 2004). These youth may moreover be at a heightened risk of exclusionary school discipline, which can drive them into more delinquent peer groups (Jacobsen, 2020) and inadvertently counter the anticipated violence-reducing benefits of a positive climate (Mowen et al., 2020). All these network processes may furthermore shape violence perpetration differently by gender or for sexual and racial minorities, for whom sanctioning and isolation are often disproportionately experienced (Donoghue, 2022). Finally, it is important to note that the pathways through which school-level positive climate and friendship network density shape violence perpetration may vary by specific types of violence and who they are perpetrated against. For example, severe forms of violence among students of a highly cohesive school may be acceptable only for males when perpetrated against students attending a rival school (Gould 2003), while only less severe forms of violence may acceptable among female friends (Kreager, 2007). In sum, more research on the interplay between school social organization and youth network processes is necessary to enlighten the present findings and the broader literature on schools and youth well-being.

The present results and recommendations for research could also shed light on the limited success of community social disorganization processes more broadly—including dense ties and informal social control norms in neighborhoods—in explaining inequalities in adolescent violence and delinquency (Browning et al., 2016). Specifically, the present findings suggest that school and neighborhood informal control processes are least likely to reduce youth violence when these settings are also characterized by dense youth friendship ties. One particularly fruitful path forward could be to focus on the informal control capacity of teachers rather than that of students and their parents (Lösel & Farrington, 2012). For example, research finds that schools characterized by high levels of “relational trust” among school staff are better equipped to seek and adopt innovative learning approaches, feel more responsible for student success, and collaborate with parents to maximize students’ social and academic well-being (Bryk & Schneider, 2002). Indeed, one study found that adolescents were less likely to be arrested or suspended when attending schools where teachers reported higher levels of “school collective efficacy,” captured with measures of the extent of teacher-teacher trust and collective responsibility for student learning (Kirk, 2009). Research considering how neighborhood and school sources of social capital may interact, such as was suggested by the success of the Harlem Childrens’ Zone (Putnam, 2015), may also enlighten the potential for neighborhood and school social processes to improve adolescent behavior, but remains under-examined. Finally, it should be noted that dimensions of school social organization may have lagged effects, such that their consequences are not evident until youth enter adulthood (Payne & Welch, 2016). For example, while this study found a nonsignificant association of school-level pupil-teacher ratio with violence in adolescence, other research suggests that exposure to lower pupil-teacher ratios in schools can reduce students’ risk of becoming incarcerated as adults (Arum & LaFree, 2008).

It is important to consider the limitations of this study, particularly given the policy relevance of positive school climate. The Add Health study began in 1994-1995 and has been extensively used to examine consequences of school experiences for adolescent violence on a national scale. The ability to consider interactions between measures of positive school-level climate and network density made this dataset extraordinarily well-suited to test the present hypotheses. However, a different pattern of findings might be found when considering more recent cohorts of youth. For example, the decline of neighborhood schools could make school experiences less relevant to violence in contemporary cohorts. Schools have also undergone notable organizational changes since the Add Health study began, and the adolescent social sphere is increasingly shifting to online platforms (e.g., social media), all of which could reduce the relevance of school-level processes for understanding youth behavior (Wilson et al., 2022). Consistent with most studies of school climate, the non-experimental nature of these analyses renders them vulnerable to selection effects. The influence of selection could be partially captured and reduced with the control variables, but many selection processes cannot be observed or accounted for with these data. Furthermore, positive school-level climate and friendship network density may be heavily shaped by out-of-school factors, such as those in the local community or district-level initiatives (Arum, 2000). Associations of school-level variables could thus be partially capturing effects of processes occurring beyond the school, but which may be magnified when youth are at school—the most routinely inhabited environment beyond the home for the vast majority of youth (Pinchak et al., 2022). Ultimately, more data on social processes occurring in youths’ schools, neighborhoods, and on- and offline activity spaces remains necessary to fully inform how features of adolescents’ schools, specifically, shape their behavior.

## Conclusion

Over three decades of research have investigated whether youth attending schools with a more positive climate perpetrate less violence. This study considered how this association varies across schools, focusing specifically on the school-level density of student friendship ties. Results indicate that the violence-reducing association of positive school climate is only evident among schools with a low density of student friendship ties, and, strikingly, that a more positive school climate is associated with increased violence among youth attending schools with a high density of student friendship ties. These findings underscore that theories and initiatives proposing to reduce violence and increase informal controls by fostering cohesion in adolescents’ contexts should pay careful attention to how network processes may limit returns, such as by exacerbating youths’ conflicts and bolstering efforts to subvert control of their behavior.

## Supplementary information

Appendix

## Footnotes

1 Coleman also hypothesized benefits of schools having more “intergenerational closure,” or the degree to which students’ parents know one another and their children’s friends. Studies have found little evidence supporting this hypothesis, however (Morgan & Todd, 2009).

2 Violence scores generated from two-parameter item response models were also considered. This approach yielded scores highly correlated with those based on the Rasch approach, however (=0.99 for Wave I violence scores, and 0.97 for Wave II violence scores).

3 Students were also asked about their perceptions of safety at school, but these are excluded because this is often considered more of an outcome than a component of positive school climate (Bradshaw et al., 2021).

4 This coefficient can be interpreted similarly to a Cronbach’s alpha, where values closer to 1 indicate greater reliability. For reference, adequate school-level reliabilities based on social surveys of students and teachers tend to range from 0.6 to 0.8 (e.g., Lynch et al., 2013).

5 Specifically, the Add Health Network Variables Codebook authors state the following: “Because we wish to provide substantively useful variables, only schools in which more than 50 percent of the student body completed the questionnaire are used to calculate the network measures described here. Relying on global network characteristics from schools with incomplete data would provide misleading images of the school’s social structure” (Carolina Population Center, University of North Carolina, 2001, p. 1).

6 Add Health analysts are instructed to “Choose a single-level model and single-level weight if you are only interested in including school-level variables as covariates but not in obtaining variance components estimates (i.e., random effects)” (Chen & Harris, 2020, p. 53) which aligns with the goals of this study (i.e., hypothesis testing). These “single-level” models are known as population average models, and have been advocated in the Add Health analysis guidelines as well as in work addressing how to appropriately model Add Health contextual data (Christ, 2014), where this approach is discussed as employing “standard error estimators that are robust to clustering.” Here the provision of weights at the individual- and school-level by the Add Health team are also discussed, although standard errors estimated when using multilevel models with these weights are “less efficient” than those estimated from models utilizing the single-level weights (Christ, 2014). Add Health does not provide weights for census tract-level variables because these were not part of the sampling design. Finally, it is important to note that many conventional model fit statistics—such as R-squared, BIC, AIC, and incremental F tests—are invalid or cannot be estimated for these models, particularly when employing multiple imputation (Williams, 2021).

7 In the models replacing positive school climate with school community attachment, the VIF for this measure is 1.98 and 2.73 for relative network density. When used in the reduced control variables models, these VIFs decline to 1.78 and 1.76, respectively. In the models replacing positive school climate with low interpersonal trouble, the VIF for this measure is 2.91 and 2.97 for relative network density. When used in the reduced control variables models, these VIFs decline to 2.02 and 1.86, respectively.
