# Supplementary material for: A Paradox of School Social Organization: Positive School Climate, Friendship Network Density, and Adolescent Violence
Source: J Youth Adolesc. 2024 Jun 24;53(11):2623–41. doi: 10.1007/s10964-024-02034-2 (PMC11467121; doi:10.1007/s10964-024-02034-2)

**Appendix**

**Supplemental Descriptive Statistics**

**Appendix Table 1.** Sample Size and Proportions for Violence Items.

**Appendix Table 2.** Descriptive Statistics for Wave I Parent Relationship and Impulsivity Items.

**Appendix Figure 1.** Histogram of Wave II Violence Scores.

**Appendix Figure 2.** Joint Distributions of z-score Standardized School-level Positive Climate and Relative Network Density for the 113 Schools in the Analytic Sample.

**Tables and Figures for Presented Models**

**Appendix Table 3.** Full Tables of Coefficients and Standard Errors from the Presented Models.

**Appendix Figure 3.** Presented Model 8: Average Marginal Effects (AME) of School Community Attachment and Relative Network Density at Percentiles of One Another and Predicted Values.

**Tables and Figures for Reduced Control Variables Models**

**Appendix Table 4.** Full Tables of Coefficients and Standard Errors from Reduced Controls Models.

**Appendix Figure 4.** Reduced Controls Models: Average Marginal Effects (AME) of Positive School Climate Measures and Relative Network Density at Percentiles of One Another and Predicted Values.

**Tables and Figures for Models with Sex Interactions**

**Appendix Table 5.** Full Tables of Coefficients and Standard Errors from Models with Sex Interactions.

**Appendix Figure 5.** Linear and Poisson Models with Sex Interactions: Average Marginal Effects (AME) of Positive School Climate and Relative Network Density at Percentiles of One Another by Respondent Sex.

**Appendix Figure 6.** Linear and Poisson Models with Sex Interactions: Average Marginal Effects (AME) of Low Interpersonal Trouble and Relative Network Density at Percentiles of One Another by Respondent Sex.

**Appendix Figure 7.** Linear and Poisson Models with Sex Interactions: Average Marginal Effects (AME) of School Community Attachment and Relative Network Density at Percentiles of One Another by Respondent Sex.

**Tables and Figures for Models with Respondent Network Interactions**

**Appendix Table 6.** Full Tables of Coefficients and Standard Errors from Models with Respondent Network Interactions.

**Appendix Figure 8.** Respondent Network Interaction Models: Average Marginal Effects (AME) of Positive School Climate and Relative Network Density at Percentiles of One Another.

**Appendix Figure 9.** Respondent Network Interaction Models: Average Marginal Effects (AME) of Low Interpersonal Trouble and Relative Network Density at Percentiles of One Another.

**Appendix Figure 10.** Respondent Network Interaction Models: Average Marginal Effects (AME) of School Community Attachment and Relative Network Density at Percentiles of One Another.

| Appendix Table 1. Sample Size and Proportions for Violence Items. | | | | |
| --- | --- | --- | --- | --- |
|  | Full Sample | | Analytic Sample | |
|  | N | Mean | N | Mean |
| *Wave I violence items* |  |  |  |  |
| Participated in a physical fight | 20,551 | 0.323 | 11,680 | 0.326 |
| Hurt someone badly in a fight | 20,547 | 0.188 | 11,680 | 0.187 |
| Participated in a group fight | 20,561 | 0.200 | 11,689 | 0.207 |
| Carried a weapon to school | 20,566 | 0.057 | 11,691 | 0.054 |
| Threatened or used a weapon to get something | 20,568 | 0.043 | 11,690 | 0.046 |
| Pulled a weapon on someone | 20,578 | 0.049 | 11,697 | 0.047 |
| Shot or stabbed someone | 20,568 | 0.020 | 11,694 | 0.019 |
| *Wave II violence items* |  |  |  |  |
| Participated in a physical fight | 14,672 | 0.198 | 11,722 | 0.197 |
| Hurt someone badly in a fight | 14,715 | 0.081 | 11,753 | 0.081 |
| Participated in a group fight | 14,668 | 0.181 | 11,718 | 0.180 |
| Carried a weapon to school | 14,738 | 0.037 | 11,771 | 0.036 |
| Threatened or used a weapon to get something | 14,667 | 0.036 | 11,718 | 0.035 |
| Pulled a weapon on someone | 14,674 | 0.046 | 11,723 | 0.044 |
| Shot or stabbed someone | 14,678 | 0.018 | 11,724 | 0.017 |

| Appendix Table 2. Descriptive Statistics for Wave I Parent Relationship and Impulsivity Items. | | | | |  |
| --- | --- | --- | --- | --- | --- |
| Variable Names | N | Mean | *SD* | Range |  |
| **Parent Relationship Quality (alpha = .87)** | | | | |  |
| *Mother Relationship Perceptions* |  |  |  |  |  |
| Mother helps you understand what went wrong. | 19,447 | 4.10 | *0.91* | 1 - 5 |  |
| Mother encourages you to be independent. | 19,432 | 4.18 | *0.88* | 1 - 5 |  |
| Mother high school attainment expectations. | 19,380 | 4.72 | *0.86* | 1 - 5 |  |
| Mother closeness. | 19,469 | 4.52 | *0.80* | 1 - 5 |  |
| Mother cares about you. | 19,463 | 4.85 | *0.50* | 1 - 5 |  |
| Mother communication. | 19,453 | 4.03 | *1.02* | 1 - 5 |  |
| Mother warmth. | 19,444 | 4.35 | *0.81* | 1 - 5 |  |
| *Father Relationship Perceptions* |  |  |  |  |  |
| Father high school attainment expectations. | 14,378 | 4.69 | *0.89* | 1 - 5 |  |
| Father closeness. | 14,442 | 4.24 | *0.98* | 1 - 5 |  |
| Father cares about you. | 14,435 | 4.73 | *0.65* | 1 - 5 |  |
| Father communication. | 14,422 | 3.91 | *1.05* | 1 - 5 |  |
| Father warmth. | 14,424 | 4.11 | *0.93* | 1 - 5 |  |
| *"Family" or "Parents" Relationship Perceptions* |  |  |  |  |  |
| Family pays attention to you. | 20,590 | 3.91 | *0.94* | 1 - 5 |  |
| Family is fun. | 20,588 | 3.71 | *1.03* | 1 - 5 |  |
| Family understands you. | 20,615 | 3.58 | *1.03* | 1 - 5 |  |
| Parents care about you. | 20,608 | 4.78 | *0.58* | 1 - 5 |  |
| **Impulsivity (alpha = .74)** |  |  |  |  |  |
| Reversed: [When problem solving] you get as many facts about the problem as possible. | 20,562 | 2.17 | *0.86* | 1 - 5 |  |
| Reversed: You think of as many different ways to approach the problem as possible. | 20,565 | 2.03 | *0.77* | 1 - 5 |  |
| Reversed: Use a systematic method for judging and comparing alternatives. | 20,479 | 2.38 | *0.88* | 1 - 5 |  |
| Reversed: [After problem solving], you usually try to analyze what went right and what went wrong. | 20,569 | 2.18 | *0.82* | 1 - 5 |  |
| Notes: All items are recoded such that 1 = strongly disagree and 5 = strongly agree. Alphas calculated with pairwise correlations. Questions for mother help with understanding what went wrong and encouragement of independence are not asked for fathers. Due to significant missingness across parent questions, items were combined using a two-level hierarchical linear model with questions clustered within respondents, and the estimated respondent-level random effect is used as the measure of parent relationship quality. | | | | |  |
|  |  |  |  |  |  |
|  |  |  |  |  |  |

Appendix Figure 1. Histogram of Wave II Violence Scores.


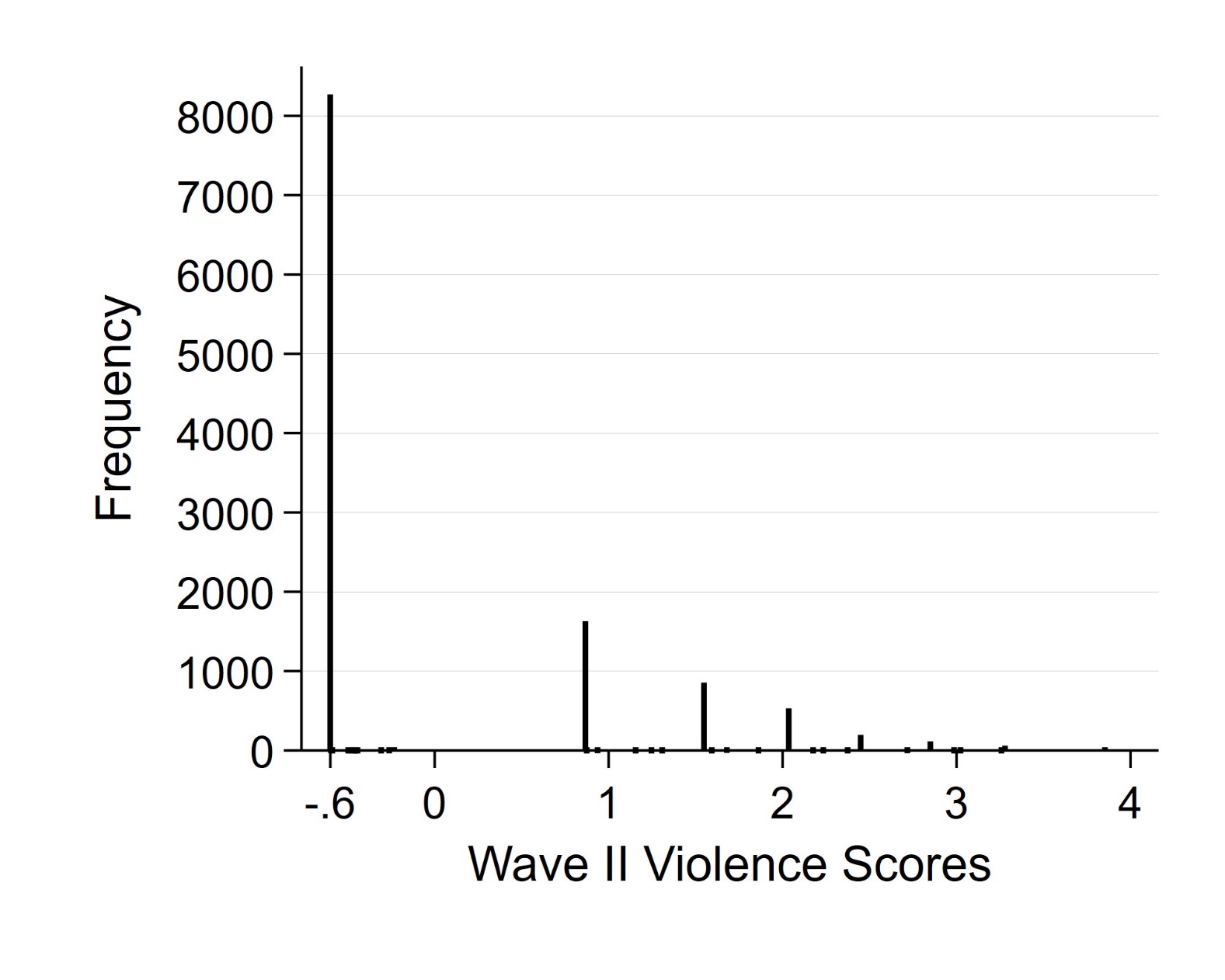


Appendix Figure 2. Joint Distributions of z-score Standardized School-level Positive Climate and Relative Network Density for the 113 Schools in the Analytic Sample.


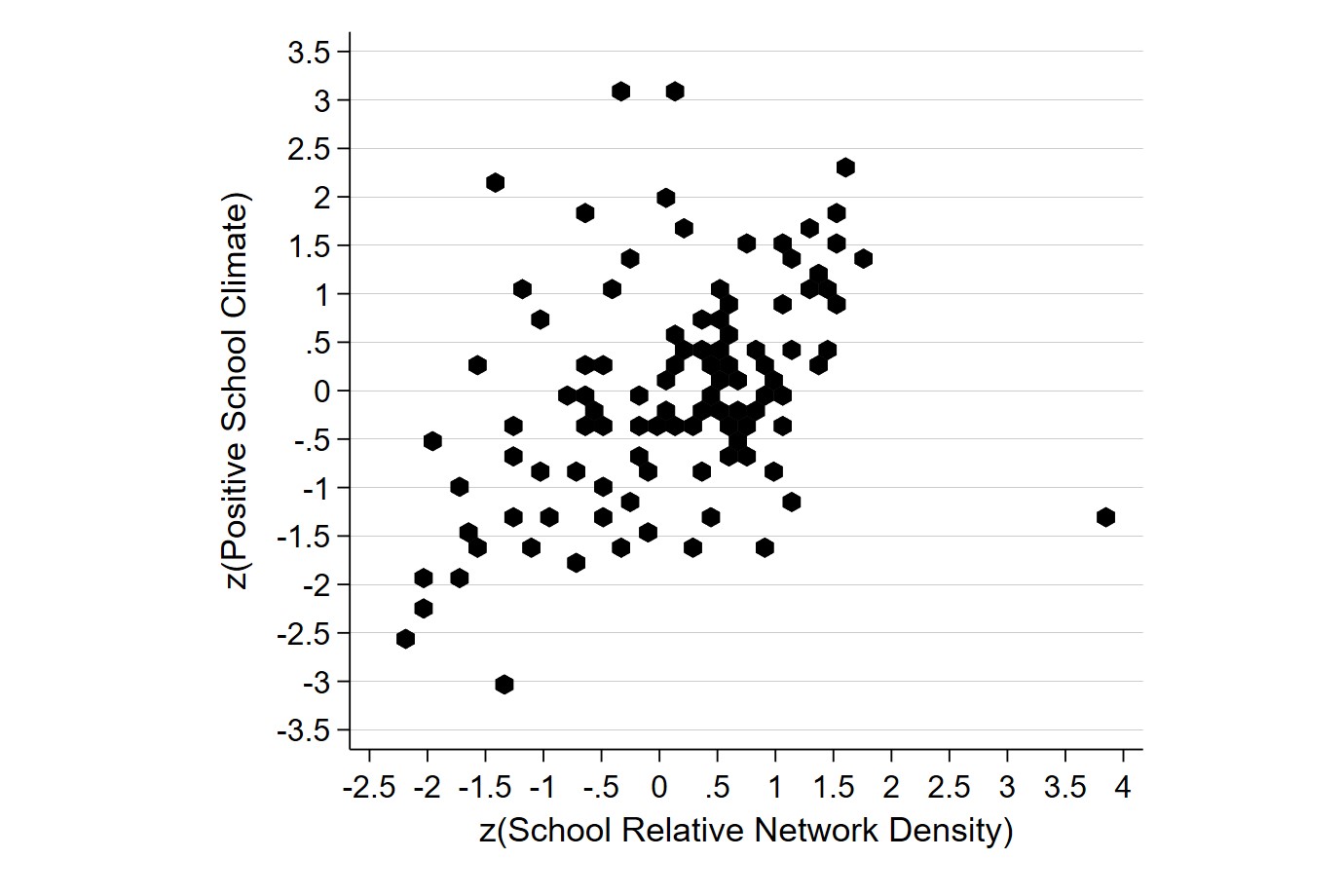


Notes: Positive School Climate 5^th^, 10^th^, 25^th^, 50^th^, 75^th^, 90^th^ and 95^th^ percentiles correspond to values of -1.70, -1.27, -.35, -.05, .43, 1.35, and 1.70, respectively. School Relative Network Density 5^th^, 10^th^, 25^th^, 50^th^, 75^th^, 90^th^ and 95^th^ percentiles correspond to values of -1.61, -1.32, -1.01, .24, .76, 1.11, and 1.39, respectively. These percentiles of relative network density in its raw metric (i.e., not standardized) correspond to values of .25, .28, .32, .45, .50, .54, and .57, respectively. Results are robust to the exclusion of the school with a relative network density of 3.89 (student n = 18 in the analytic sample).

| Appendix Table 3. Full Tables of Coefficients and Standard Errors from the Presented Models. | | | | | | |  |  |  |
| --- | --- | --- | --- | --- | --- | --- | --- | --- | --- |
|  | 1 | 2 | 3 | 4 | 5 | 6 | 7 | 8 |  |
| Individual-level Controls |  |  |  |  |  |  |  |  |  |
| Age | -0.025 | -0.024 | -0.022 | -0.035 | -0.040 | -0.041 | -0.032 | -0.017 |  |
|  | (0.117) | (0.117) | (0.117) | (0.116) | (0.100) | (0.100) | (0.102) | (0.101) |  |
| Age^2 | -0.001 | -0.001 | -0.001 | -0.001 | 0.000 | 0.000 | 0.000 | -0.000 |  |
|  | (0.004) | (0.004) | (0.004) | (0.004) | (0.003) | (0.003) | (0.003) | (0.003) |  |
| Female | -0.306*** | -0.305*** | -0.305*** | -0.305*** | -0.134*** | -0.135*** | -0.135*** | -0.134*** |  |
|  | (0.024) | (0.024) | (0.024) | (0.024) | (0.023) | (0.023) | (0.023) | (0.023) |  |
| Race |  |  |  |  |  |  |  |  |  |
| White (ref.) | - | - | - | - | - | - | - | - |  |
| Black | 0.023 | 0.024 | 0.023 | 0.021 | -0.062 | -0.059 | -0.063 | -0.058 |  |
|  | (0.056) | (0.055) | (0.056) | (0.056) | (0.047) | (0.047) | (0.048) | (0.047) |  |
| Hispanic | 0.109* | 0.109* | 0.108* | 0.107* | 0.053 | 0.061 | 0.059 | 0.061 |  |
|  | (0.052) | (0.052) | (0.052) | (0.052) | (0.048) | (0.048) | (0.048) | (0.048) |  |
| Asian | -0.088 | -0.090 | -0.091 | -0.084 | -0.067 | -0.062 | -0.062 | -0.064 |  |
|  | (0.063) | (0.062) | (0.063) | (0.067) | (0.054) | (0.054) | (0.054) | (0.053) |  |
| Other race/ethnicity | 0.165* | 0.165* | 0.165* | 0.171* | 0.107+ | 0.100+ | 0.099+ | 0.099+ |  |
|  | (0.068) | (0.068) | (0.068) | (0.069) | (0.057) | (0.057) | (0.058) | (0.057) |  |
| Resides with two biological parents | -0.054* | -0.054* | -0.054* | -0.052* | -0.033 | -0.033 | -0.033 | -0.033 |  |
|  | (0.023) | (0.023) | (0.023) | (0.023) | (0.022) | (0.022) | (0.022) | (0.022) |  |
| Family socioeconomic status | 0.005 | 0.004 | 0.005 | 0.006 | 0.012 | 0.011 | 0.011 | 0.012 |  |
|  | (0.013) | (0.013) | (0.013) | (0.014) | (0.012) | (0.012) | (0.012) | (0.012) |  |
| Parent relationship quality | -0.100*** | -0.101*** | -0.101*** | -0.101*** | -0.046*** | -0.042** | -0.042** | -0.042** |  |
|  | (0.015) | (0.015) | (0.015) | (0.015) | (0.013) | (0.013) | (0.013) | (0.013) |  |
| Neighborhood monitoring | 0.003 | 0.003 | 0.004 | 0.004 | -0.009 | -0.009 | -0.009 | -0.009 |  |
|  | (0.014) | (0.014) | (0.014) | (0.014) | (0.012) | (0.011) | (0.011) | (0.011) |  |
| Impulsivity | 0.033* | 0.032* | 0.032* | 0.033* | 0.003 | 0.002 | 0.002 | 0.001 |  |
|  | (0.014) | (0.014) | (0.014) | (0.014) | (0.013) | (0.013) | (0.013) | (0.013) |  |
| GPA | -0.089*** | -0.090*** | -0.089*** | -0.091*** | -0.054*** | -0.048*** | -0.047*** | -0.048*** |  |
|  | (0.013) | (0.013) | (0.013) | (0.013) | (0.012) | (0.012) | (0.012) | (0.012) |  |
| Suspended | 0.446*** | 0.445*** | 0.446*** | 0.447*** | 0.188*** | 0.173*** | 0.174*** | 0.172*** |  |
|  | (0.028) | (0.028) | (0.028) | (0.028) | (0.026) | (0.026) | (0.026) | (0.026) |  |
| Personal network size | 0.004 | 0.005 | 0.005 | 0.005 | 0.002 | 0.001 | 0.001 | 0.001 |  |
|  | (0.003) | (0.003) | (0.003) | (0.003) | (0.003) | (0.003) | (0.003) | (0.003) |  |
| No network data | 0.110** | 0.110** | 0.110** | 0.109** | 0.052 | 0.045 | 0.045 | 0.045 |  |
|  | (0.036) | (0.036) | (0.036) | (0.036) | (0.033) | (0.034) | (0.034) | (0.034) |  |
| Neighborhood socioeconomic disadvantage | 0.010 | 0.010 | 0.011 | 0.016 | 0.019 | 0.019 | 0.021 | 0.017 |  |
|  | (0.020) | (0.020) | (0.020) | (0.019) | (0.017) | (0.017) | (0.018) | (0.018) |  |
| Neighborhood residential instability | 0.014 | 0.014 | 0.013 | 0.014 | 0.007 | 0.007 | 0.006 | 0.007 |  |
|  | (0.018) | (0.018) | (0.018) | (0.019) | (0.016) | (0.016) | (0.017) | (0.016) |  |
| School-level Measures |  |  |  |  |  |  |  |  |  |
| County Population density | 0.013 | 0.012 | 0.011 | 0.011 | 0.002 | 0.002 | 0.001 | 0.004 |  |
|  | (0.010) | (0.010) | (0.009) | (0.008) | (0.007) | (0.007) | (0.007) | (0.007) |  |
| School socioeconomic disadvantage | -0.004 | -0.003 | -0.006 | -0.019 | -0.033 | -0.029 | -0.031 | -0.029 |  |
|  | (0.029) | (0.029) | (0.029) | (0.029) | (0.025) | (0.025) | (0.025) | (0.024) |  |
| %Black | -0.012 | -0.012 | -0.013 | -0.013 | 0.009 | 0.006 | 0.006 | 0.004 |  |
|  | (0.021) | (0.021) | (0.021) | (0.021) | (0.018) | (0.018) | (0.019) | (0.018) |  |
| %Hispanic | 0.061+ | 0.063+ | 0.060+ | 0.062* | 0.065* | 0.063* | 0.063* | 0.062* |  |
|  | (0.032) | (0.033) | (0.032) | (0.031) | (0.029) | (0.029) | (0.029) | (0.029) |  |
| Pupil-teacher ratio | -0.001 | -0.001 | -0.001 | -0.000 | 0.001 | 0.001 | 0.001 | 0.001 |  |
|  | (0.005) | (0.005) | (0.005) | (0.005) | (0.004) | (0.004) | (0.004) | (0.004) |  |
| School suspension rate | 0.068 | 0.119 | 0.050 | 0.059 | 0.158 | 0.156 | 0.072 | 0.205 |  |
|  | (0.161) | (0.156) | (0.164) | (0.159) | (0.120) | (0.118) | (0.113) | (0.130) |  |
| Student population size | -0.000* | -0.000* | -0.000* | -0.000 | -0.000+ | -0.000 | -0.000 | -0.000 |  |
|  | (0.000) | (0.000) | (0.000) | (0.000) | (0.000) | (0.000) | (0.000) | (0.000) |  |
| Private school | -0.011 | -0.032 | -0.020 | 0.011 | -0.061 | -0.064 | -0.056 | -0.067 |  |
|  | (0.065) | (0.071) | (0.071) | (0.066) | (0.051) | (0.051) | (0.050) | (0.053) |  |
| School-level Social Processes |  |  |  |  |  |  |  |  |  |
| Positive school climate | -0.019 |  | -0.018 | -0.026 | 0.008 | 0.008 |  |  |  |
|  | (0.019) |  | (0.018) | (0.017) | (0.014) | (0.014) |  |  |  |
| Relative network density |  | -0.013 | -0.012 | 0.007 | 0.005 | 0.006 | 0.008 | 0.001 |  |
|  |  | (0.021) | (0.020) | (0.019) | (0.017) | (0.017) | (0.016) | (0.019) |  |
| Positive school climate * Network density |  |  |  | 0.033*** | 0.019* | 0.018* |  |  |  |
|  |  |  |  | (0.009) | (0.008) | (0.008) |  |  |  |
| Low Interpersonal Trouble |  |  |  |  |  |  | -0.010 |  |  |
|  |  |  |  |  |  |  | (0.017) |  |  |
| Low Interpersonal Trouble * Network density |  |  |  |  |  |  | 0.023* |  |  |
|  |  |  |  |  |  |  | (0.010) |  |  |
| School Attachment |  |  |  |  |  |  |  | 0.016 |  |
|  |  |  |  |  |  |  |  | (0.015) |  |
| School Attachment * Network density |  |  |  |  |  |  |  | 0.010 |  |
|  |  |  |  |  |  |  |  | (0.011) |  |
| Added Individual-level Variables |  |  |  |  |  |  |  |  |  |
| Wave I violence |  |  |  |  | 0.456*** | 0.447*** | 0.447*** | 0.448*** |  |
|  |  |  |  |  | (0.014) | (0.014) | (0.014) | (0.014) |  |
| Interpersonal trouble with students |  |  |  |  |  | 0.023+ | 0.022+ | 0.022+ |  |
|  |  |  |  |  |  | (0.013) | (0.013) | (0.013) |  |
| Interpersonal trouble with teachers |  |  |  |  |  | 0.028* | 0.028* | 0.028* |  |
|  |  |  |  |  |  | (0.012) | (0.012) | (0.012) |  |
| School attachment |  |  |  |  |  | 0.028+ | 0.029+ | 0.027+ |  |
|  |  |  |  |  |  | (0.015) | (0.015) | (0.015) |  |
| Teacher fairness |  |  |  |  |  | -0.026+ | -0.025+ | -0.027* |  |
|  |  |  |  |  |  | (0.013) | (0.013) | (0.013) |  |
| (Intercept) | 0.671 | 0.662 | 0.662 | 0.714 | 0.575 | 0.559 | 0.498 | 0.346 |  |
|  | (0.889) | (0.890) | (0.887) | (0.885) | (0.763) | (0.766) | (0.785) | (0.770) |  |
| N (Respondents) | 11771 | 11771 | 11771 | 11771 | 11771 | 11771 | 11771 | 11771 |  |
| N (Schools) | 113 | 113 | 113 | 113 | 113 | 113 | 113 | 113 |  |
| *** p < 0.001; ** p < 0.01; * p < 0.05; + p < 0.1. | | | | | | | | |  |
| Notes: Coefficients with errors in parentheses. All continuous independent variables are z-score standardized (mean = 0, SD = 1) except for county population density, pupil-teacher ratio, school suspension rate, personal network size, age, and school student population. Models are weighted according to Add Health Guidelines using the Stata "svyset" command. | | | | | | | | |  |
|  |  |  |  |  |  |  |  |  |  |
|  |  |  |  |  |  |  |  |  |  |
|  |  |  |  |  |  |  |  |  |  |

Appendix Figure 3. Presented Model 8: Average Marginal Effects (AME) of School Community Attachment and Relative Network Density at Percentiles of One Another and Predicted Values.


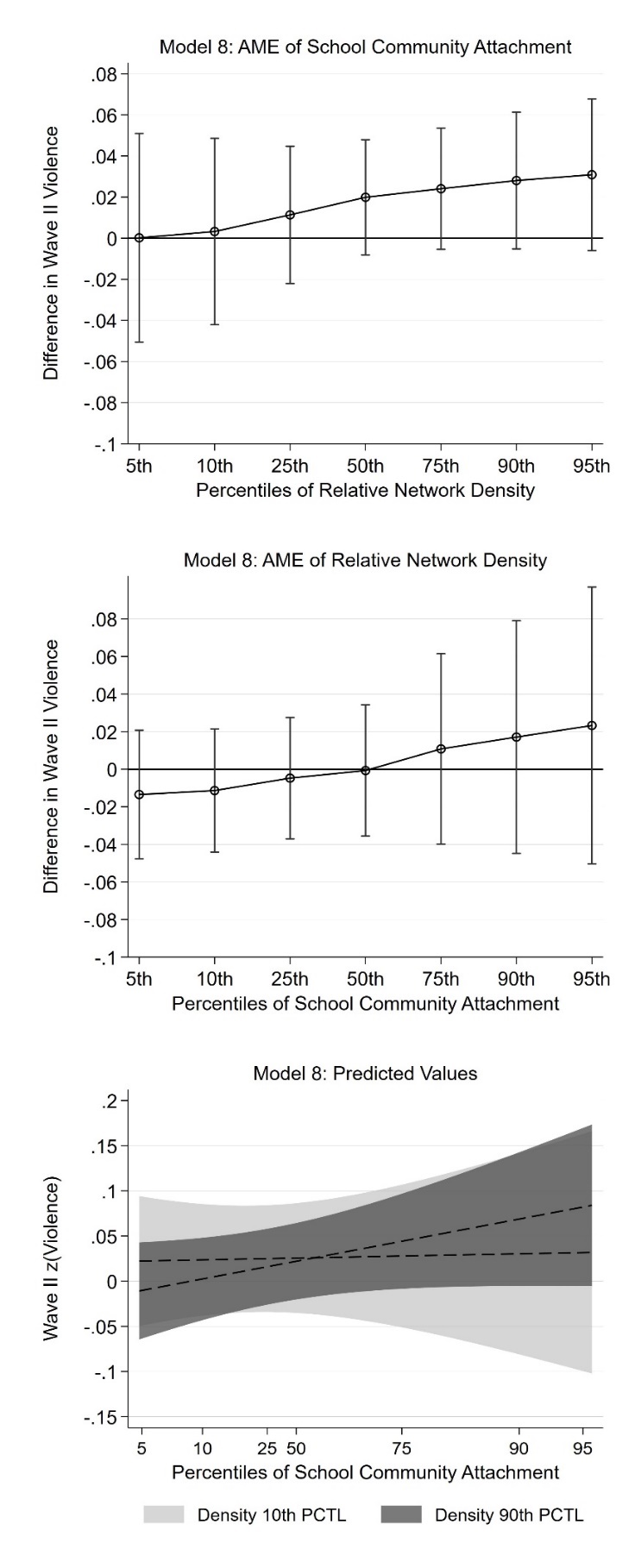


| Appendix Table 4. Full Tables of Coefficients and Standard Errors from Reduced Controls Models. | | | |  |  |
| --- | --- | --- | --- | --- | --- |
|  | Reduced Controls | Low Trouble, Reduced | Comm. Attach., Reduced | |  |
|  |  |  |  |  |  |
| Individual-level Controls |  |  |  | |  |
| Age | -0.057 | -0.037 | -0.035 | |  |
|  | (0.103) | (0.102) | (0.101) | |  |
| Age^2 | 0.001 | 0.000 | 0.000 | |  |
|  | (0.003) | (0.003) | (0.003) | |  |
| Female | -0.135*** | -0.135*** | -0.134*** | |  |
|  | (0.023) | (0.023) | (0.023) | |  |
| Race |  |  |  | |  |
| White (ref.) | - | - | - | |  |
| Black | -0.058 | -0.060 | -0.057 | |  |
|  | (0.036) | (0.036) | (0.036) | |  |
| Hispanic | 0.107* | 0.105* | 0.109* | |  |
|  | (0.044) | (0.044) | (0.044) | |  |
| Asian | -0.046 | -0.047 | -0.050 | |  |
|  | (0.051) | (0.052) | (0.050) | |  |
| Other race/ethnicity | 0.115+ | 0.114+ | 0.114+ | |  |
|  | (0.059) | (0.060) | (0.058) | |  |
| Resides with two biological parents | -0.032 | -0.031 | -0.032 | |  |
|  | (0.022) | (0.022) | (0.022) | |  |
| Family socioeconomic status | 0.013 | 0.013 | 0.013 | |  |
|  | (0.012) | (0.012) | (0.012) | |  |
| Parent relationship quality | -0.042** | -0.043** | -0.042** | |  |
|  | (0.013) | (0.013) | (0.013) | |  |
| Neighborhood monitoring | -0.009 | -0.009 | -0.009 | |  |
|  | (0.011) | (0.011) | (0.011) | |  |
| Impulsivity | 0.001 | 0.001 | 0.000 | |  |
|  | (0.013) | (0.013) | (0.013) | |  |
| GPA | -0.047*** | -0.047*** | -0.047*** | |  |
|  | (0.012) | (0.012) | (0.012) | |  |
| Suspended | 0.174*** | 0.174*** | 0.172*** | |  |
|  | (0.026) | (0.026) | (0.026) | |  |
| Personal network size | 0.001 | 0.001 | 0.001 | |  |
|  | (0.003) | (0.003) | (0.003) | |  |
| No network data | 0.043 | 0.044 | 0.044 | |  |
|  | (0.034) | (0.034) | (0.034) | |  |
| Neighborhood socioeconomic disadvantage | 0.016 | 0.016 | 0.014 | |  |
|  | (0.018) | (0.018) | (0.018) | |  |
| Neighborhood residential instability | 0.014 | 0.014 | 0.014 | |  |
|  | (0.016) | (0.016) | (0.016) | |  |
| School-level Measures |  |  |  | |  |
| County Population density |  |  |  | |  |
|  |  |  |  | |  |
| School socioeconomic disadvantage |  |  |  | |  |
|  |  |  |  | |  |
| %Black |  |  |  | |  |
|  |  |  |  | |  |
| %Hispanic |  |  |  | |  |
|  |  |  |  | |  |
| Pupil-teacher ratio | 0.003 | 0.003 | 0.002 | |  |
|  | (0.004) | (0.004) | (0.004) | |  |
| School suspension rate | 0.040 | -0.038 | 0.108 | |  |
|  | (0.124) | (0.108) | (0.127) | |  |
| Student population size |  |  |  | |  |
|  |  |  |  | |  |
| Private school |  |  |  | |  |
|  |  |  |  | |  |
| School-level Social Processes |  |  |  | |  |
| Positive school climate | 0.002 |  |  | |  |
|  | (0.013) |  |  | |  |
| Relative network density | 0.009 | 0.012 | 0.004 | |  |
|  | (0.017) | (0.016) | (0.018) | |  |
| Low interpersonal trouble |  | -0.016 |  | |  |
|  |  | (0.014) |  | |  |
| School community attachment |  |  | 0.014 | |  |
|  |  |  | (0.014) | |  |
| Interactions |  |  |  | |  |
| Positive school climate * Network density | 0.020* |  |  | |  |
|  | (0.008) |  |  | |  |
| Low interpersonal trouble * Network density |  | 0.024* |  | |  |
|  |  | (0.011) |  | |  |
| School community attachment * Network density |  |  | 0.012 | |  |
|  |  |  | (0.012) | |  |
| Added Individual-level Variables |  |  |  | |  |
| Wave I violence | 0.447*** | 0.447*** | 0.448*** | |  |
|  | (0.014) | (0.014) | (0.014) | |  |
| Interpersonal trouble with students | 0.023+ | 0.023+ | 0.022+ | |  |
|  | (0.013) | (0.013) | (0.013) | |  |
| Interpersonal trouble with teachers | 0.028* | 0.028* | 0.028* | |  |
|  | (0.012) | (0.012) | (0.012) | |  |
| School attachment | 0.028+ | 0.029+ | 0.027+ | |  |
|  | (0.015) | (0.015) | (0.015) | |  |
| Teacher fairness | -0.027* | -0.026* | -0.028* | |  |
|  | (0.013) | (0.013) | (0.013) | |  |
| (Intercept) | 0.627 | 0.471 | 0.432 | |  |
|  | (0.786) | (0.785) | (0.773) | |  |
| N (Respondents) | 11771 | 11771 | 11771 | |  |
| N (Schools) | 113 | 113 | 113 | |  |
| *** p < 0.001; ** p < 0.01; * p < 0.05; + p < 0.1. | | | |  |  |
| Notes: Coefficients with errors in parentheses. All continuous independent variables are z-score standardized (mean = 0, SD = 1) except for county population density, pupil-teacher ratio, school suspension rate, personal network size, age, and school student population. Models are weighted according to Add Health Guidelines using the Stata "svyset" command. | | | |  |  |
|  |  |  |  |  | |
|  |  |  |  |  | |
|  |  |  |  |  | |

Appendix Figure 4. Reduced Controls Models: Average Marginal Effects (AME) of Positive School Climate Measures and Relative Network Density at Percentiles of One Another and Predicted Values.


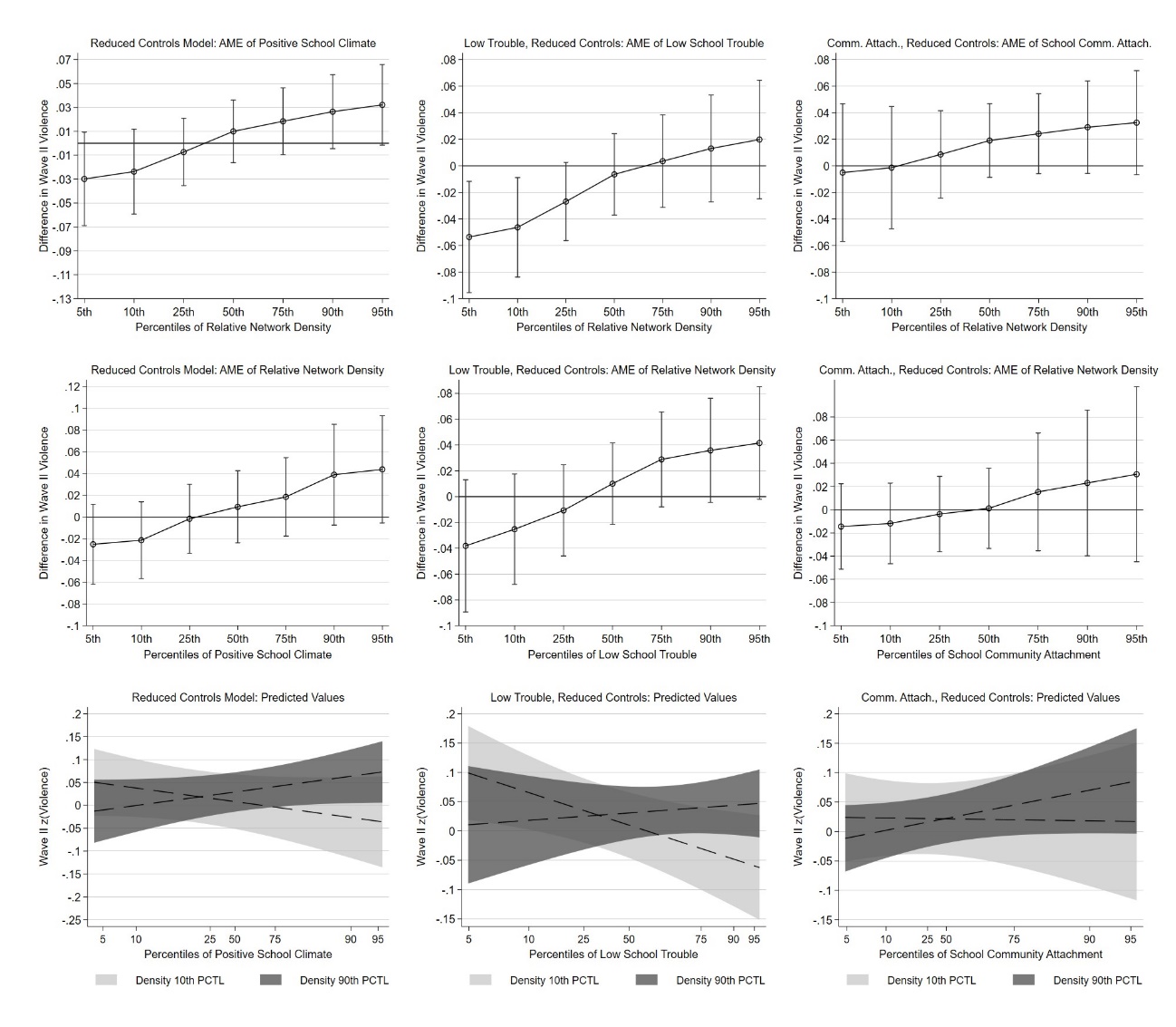


| Appendix Table 5. Full Tables of Coefficients and Standard Errors from Models with Sex Interactions. | | | | | | |  |  |
| --- | --- | --- | --- | --- | --- | --- | --- | --- |
|  | Linear | Poisson | Linear, Low Trouble | Poisson, Low Trouble | Linear, Comm. Attach. | Poisson, Comm. Attach. |  |  |
|  |  |  |  |  |  |  |  |  |
| Individual-level Controls |  |  |  |  |  |  |  |  |
| Age | -0.042 | -0.207 | -0.028 | -0.150 | -0.016 | -0.173 |  |  |
|  | (0.101) | (0.157) | (0.102) | (0.154) | (0.101) | (0.155) |  |  |
| Age^2 | 0.000 | 0.005 | 0.000 | 0.003 | -0.000 | 0.004 |  |  |
|  | (0.003) | (0.005) | (0.003) | (0.005) | (0.003) | (0.005) |  |  |
| Female | -0.136*** | -0.301*** | -0.135*** | -0.313*** | -0.139*** | -0.284*** |  |  |
|  | (0.024) | (0.046) | (0.024) | (0.048) | (0.024) | (0.044) |  |  |
| Race |  |  |  |  |  |  |  |  |
| White (ref.) | - | - | - | - | - | - |  |  |
| Black | -0.059 | -0.027 | -0.062 | -0.031 | -0.057 | -0.025 |  |  |
|  | (0.047) | (0.068) | (0.048) | (0.069) | (0.047) | (0.068) |  |  |
| Hispanic | 0.060 | 0.095 | 0.059 | 0.089 | 0.062 | 0.102 |  |  |
|  | (0.048) | (0.070) | (0.048) | (0.070) | (0.048) | (0.070) |  |  |
| Asian | -0.061 | -0.095 | -0.063 | -0.100 | -0.064 | -0.100 |  |  |
|  | (0.054) | (0.122) | (0.054) | (0.121) | (0.053) | (0.120) |  |  |
| Other race/ethnicity | 0.100+ | 0.139+ | 0.100+ | 0.138+ | 0.100+ | 0.134+ |  |  |
|  | (0.057) | (0.078) | (0.058) | (0.078) | (0.057) | (0.079) |  |  |
| Resides with two biological parents | -0.032 | -0.037 | -0.032 | -0.038 | -0.033 | -0.037 |  |  |
|  | (0.022) | (0.036) | (0.022) | (0.036) | (0.022) | (0.035) |  |  |
| Family socioeconomic status | 0.012 | -0.008 | 0.012 | -0.007 | 0.012 | -0.008 |  |  |
|  | (0.012) | (0.018) | (0.012) | (0.019) | (0.012) | (0.019) |  |  |
| Parent relationship quality | -0.042** | -0.066*** | -0.042** | -0.066*** | -0.042** | -0.066*** |  |  |
|  | (0.013) | (0.018) | (0.013) | (0.018) | (0.013) | (0.018) |  |  |
| Neighborhood monitoring | -0.009 | -0.012 | -0.009 | -0.012 | -0.009 | -0.014 |  |  |
|  | (0.011) | (0.018) | (0.011) | (0.018) | (0.011) | (0.018) |  |  |
| Impulsivity | 0.002 | 0.001 | 0.002 | 0.002 | 0.001 | 0.002 |  |  |
|  | (0.013) | (0.022) | (0.013) | (0.022) | (0.013) | (0.022) |  |  |
| GPA | -0.048*** | -0.083*** | -0.048*** | -0.083*** | -0.048*** | -0.083*** |  |  |
|  | (0.012) | (0.018) | (0.012) | (0.018) | (0.012) | (0.018) |  |  |
| Suspended | 0.173*** | 0.221*** | 0.173*** | 0.215*** | 0.171*** | 0.217*** |  |  |
|  | (0.027) | (0.036) | (0.027) | (0.036) | (0.026) | (0.035) |  |  |
| Personal network size | 0.001 | 0.001 | 0.001 | 0.001 | 0.001 | 0.001 |  |  |
|  | (0.003) | (0.005) | (0.003) | (0.005) | (0.003) | (0.005) |  |  |
| No network data | 0.044 | 0.053 | 0.044 | 0.054 | 0.045 | 0.054 |  |  |
|  | (0.034) | (0.049) | (0.034) | (0.049) | (0.034) | (0.049) |  |  |
| Neighborhood socioeconomic disadvantage | 0.019 | 0.025 | 0.021 | 0.030 | 0.018 | 0.022 |  |  |
|  | (0.017) | (0.025) | (0.018) | (0.026) | (0.018) | (0.025) |  |  |
| Neighborhood residential instability | 0.008 | 0.017 | 0.006 | 0.013 | 0.007 | 0.017 |  |  |
|  | (0.017) | (0.025) | (0.017) | (0.025) | (0.017) | (0.025) |  |  |
| School-level Measures |  |  |  |  |  |  |  |  |
| County Population density | 0.002 | 0.005 | 0.001 | 0.004 | 0.004 | 0.007 |  |  |
|  | (0.007) | (0.008) | (0.007) | (0.008) | (0.007) | (0.008) |  |  |
| School socioeconomic disadvantage | -0.029 | -0.060 | -0.030 | -0.070+ | -0.028 | -0.061 |  |  |
|  | (0.025) | (0.040) | (0.025) | (0.042) | (0.024) | (0.039) |  |  |
| %Black | 0.006 | 0.006 | 0.006 | 0.002 | 0.004 | 0.004 |  |  |
|  | (0.018) | (0.026) | (0.019) | (0.027) | (0.018) | (0.026) |  |  |
| %Hispanic | 0.064* | 0.078* | 0.063* | 0.078* | 0.062* | 0.073+ |  |  |
|  | (0.029) | (0.037) | (0.029) | (0.038) | (0.029) | (0.038) |  |  |
| Pupil-teacher ratio | 0.001 | 0.004 | 0.001 | 0.003 | 0.001 | 0.002 |  |  |
|  | (0.004) | (0.007) | (0.004) | (0.007) | (0.004) | (0.007) |  |  |
| School suspension rate | 0.158 | 0.403+ | 0.074 | 0.316 | 0.205 | 0.498* |  |  |
|  | (0.119) | (0.211) | (0.114) | (0.211) | (0.130) | (0.228) |  |  |
| Student population size | -0.000 | -0.000 | -0.000 | -0.000 | -0.000 | -0.000 |  |  |
|  | (0.000) | (0.000) | (0.000) | (0.000) | (0.000) | (0.000) |  |  |
| Private school | -0.065 | -0.147 | -0.056 | -0.121 | -0.068 | -0.130 |  |  |
|  | (0.052) | (0.096) | (0.050) | (0.093) | (0.054) | (0.099) |  |  |
| School-level Social Processes |  |  |  |  |  |  |  |  |
| Positive school climate | 0.013 | 0.045 |  |  |  |  |  |  |
|  | (0.020) | (0.028) |  |  |  |  |  |  |
| Relative network density | -0.002 | 0.011 | 0.002 | 0.025 | -0.004 | 0.018 |  |  |
|  | (0.022) | (0.032) | (0.019) | (0.028) | (0.023) | (0.033) |  |  |
| Low interpersonal trouble |  |  | 0.002 | 0.005 |  |  |  |  |
|  |  |  | (0.021) | (0.033) |  |  |  |  |
| School community attachment |  |  |  |  | 0.010 | 0.034 |  |  |
|  |  |  |  |  | (0.022) | (0.027) |  |  |
| Interactions |  |  |  |  |  |  |  |  |
| Positive school climate * Network density | 0.021+ | 0.022 |  |  |  |  |  |  |
|  | (0.011) | (0.015) |  |  |  |  |  |  |
| Low interpersonal trouble * Network density |  |  | 0.033* | 0.030 |  |  |  |  |
|  |  |  | (0.014) | (0.018) |  |  |  |  |
| School community attachment * Network density |  |  |  |  | 0.008 | 0.006 |  |  |
|  |  |  |  |  | (0.016) | (0.021) |  |  |
| Female * Positive school climate | -0.010 | -0.084+ |  |  |  |  |  |  |
|  | (0.024) | (0.048) |  |  |  |  |  |  |
| Female * Network density | 0.017 | 0.025 | 0.013 | 0.006 | 0.012 | -0.016 |  |  |
|  | (0.028) | (0.053) | (0.024) | (0.046) | (0.029) | (0.055) |  |  |
| Female * Positive school climate * Network density | -0.004 | 0.004 |  |  |  |  |  |  |
|  | (0.018) | (0.032) |  |  |  |  |  |  |
| Female * Low interpersonal trouble |  |  | -0.022 | -0.102* |  |  |  |  |
|  |  |  | (0.021) | (0.040) |  |  |  |  |
| Female * Low interpersonal trouble * Network density |  |  | -0.021 | -0.019 |  |  |  |  |
|  |  |  | (0.020) | (0.037) |  |  |  |  |
| Female * School community attachment |  |  |  |  | 0.012 | -0.004 |  |  |
|  |  |  |  |  | (0.023) | (0.041) |  |  |
| Female * School community attachment * Network density |  |  |  |  | 0.004 | 0.027 |  |  |
|  |  |  |  |  | (0.020) | (0.032) |  |  |
| Added Individual-level Variables |  |  |  |  |  |  |  |  |
| Wave I violence | 0.447*** | 0.540*** | 0.447*** | 0.537*** | 0.448*** | 0.542*** |  |  |
|  | (0.014) | (0.019) | (0.014) | (0.019) | (0.014) | (0.018) |  |  |
| Interpersonal trouble with students | 0.023+ | 0.040* | 0.023+ | 0.039* | 0.022+ | 0.039* |  |  |
|  | (0.013) | (0.016) | (0.013) | (0.016) | (0.013) | (0.016) |  |  |
| Interpersonal trouble with teachers | 0.028* | 0.026+ | 0.029* | 0.026+ | 0.029* | 0.027+ |  |  |
|  | (0.012) | (0.016) | (0.012) | (0.016) | (0.012) | (0.016) |  |  |
| School attachment | 0.028+ | 0.048* | 0.028+ | 0.047* | 0.027+ | 0.048* |  |  |
|  | (0.015) | (0.021) | (0.015) | (0.021) | (0.015) | (0.021) |  |  |
| Teacher fairness | -0.025+ | -0.029 | -0.025+ | -0.029 | -0.026* | -0.030 |  |  |
|  | (0.013) | (0.019) | (0.013) | (0.019) | (0.013) | (0.019) |  |  |
| (Intercept) | 0.563 | 1.939 | 0.476 | 1.492 | 0.338 | 1.613 |  |  |
|  | (0.768) | (1.207) | (0.785) | (1.203) | (0.769) | (1.186) |  |  |
| N (Respondents) | 11771 | 11771 | 11771 | 11771 | 11771 | 11771 |  |  |
| N (Schools) | 113 | 113 | 113 | 113 | 113 | 113 |  |  |
| *** p < 0.001; ** p < 0.01; * p < 0.05; + p < 0.1. | | | | | | |  |  |
| Notes: Coefficients with errors in parentheses. All continuous independent variables are z-score standardized (mean = 0, SD = 1) except for county population density, pupil-teacher ratio, school suspension rate, personal network size, age, and school student population. Models are weighted according to Add Health Guidelines using the Stata "svyset" command. | | | | | | |  |  |
|  | | | | | | | |  |
|  |  |  |  |  |  |  |  |  |

Appendix Figure 5. Linear and Poisson Models with Sex Interactions: Average Marginal Effects (AME) of Positive School Climate and Relative Network Density at Percentiles of One Another by Respondent Sex.


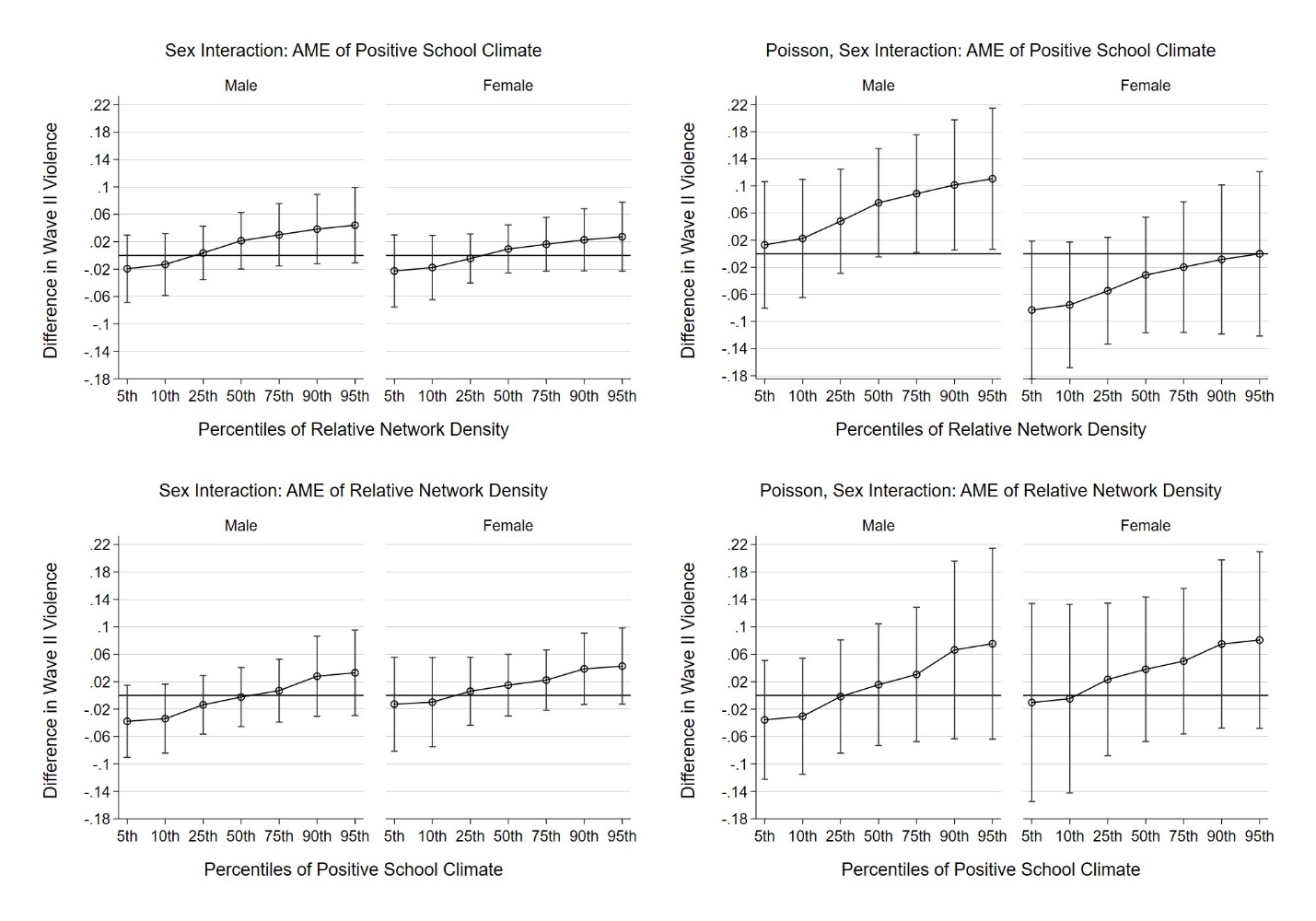


Appendix Figure 6. Linear and Poisson Models with Sex Interactions: Average Marginal Effects (AME) of Low Interpersonal Trouble and Relative Network Density at Percentiles of One Another by Respondent Sex.


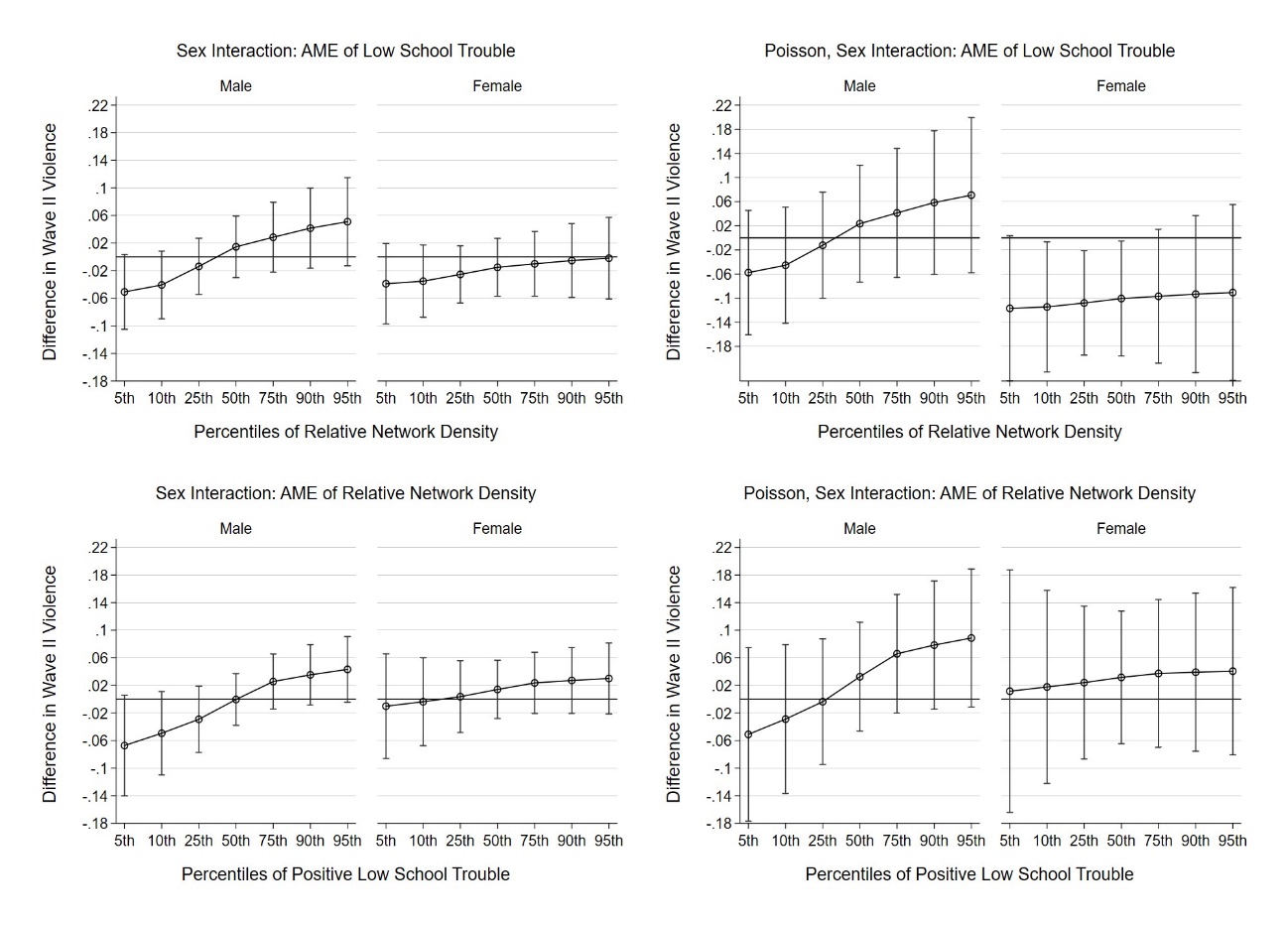


Appendix Figure 7. Linear and Poisson Models with Sex Interactions: Average Marginal Effects (AME) of School Community Attachment and Relative Network Density at Percentiles of One Another by Respondent Sex.


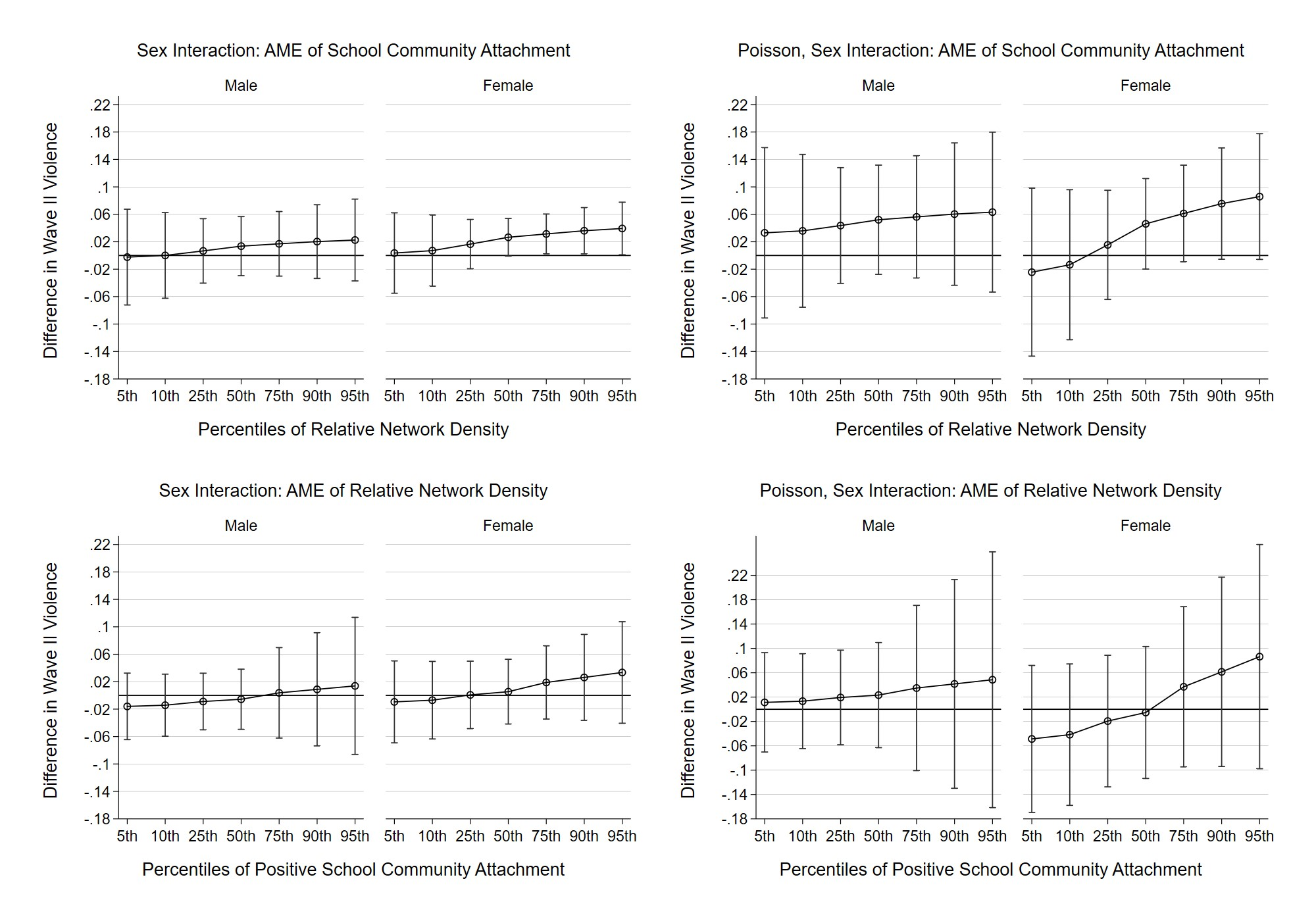


| Appendix Table 6. Full Tables of Coefficients and Standard Errors from Models with Respondent Network Interactions. | | | | | | |  |
| --- | --- | --- | --- | --- | --- | --- | --- |
|  | Network 1 | Network 2 | Network 1, Low Trouble | Network 2, Low Trouble | Network 1, Comm. Attach. | Network 2, Comm. Attach. |  |
|  |  |  |  |  |  |  |  |
| Individual-level Controls |  |  |  |  |  |  |  |
| Age | -0.040 | -0.051 | -0.026 | -0.038 | -0.019 | -0.015 |  |
|  | (0.100) | (0.100) | (0.101) | (0.101) | (0.101) | (0.101) |  |
| Age^2 | 0.000 | 0.001 | -0.000 | 0.000 | -0.000 | -0.000 |  |
|  | (0.003) | (0.003) | (0.003) | (0.003) | (0.003) | (0.003) |  |
| Female | -0.135*** | -0.133*** | -0.135*** | -0.134*** | -0.134*** | -0.133*** |  |
|  | (0.023) | (0.023) | (0.023) | (0.023) | (0.023) | (0.023) |  |
| Race |  |  |  |  |  |  |  |
| White (ref.) | - | - | - | - | - | - |  |
| Black | -0.059 | -0.057 | -0.063 | -0.061 | -0.058 | -0.055 |  |
|  | (0.047) | (0.047) | (0.048) | (0.047) | (0.047) | (0.046) |  |
| Hispanic | 0.061 | 0.061 | 0.059 | 0.060 | 0.061 | 0.063 |  |
|  | (0.048) | (0.048) | (0.048) | (0.048) | (0.048) | (0.048) |  |
| Asian | -0.062 | -0.054 | -0.063 | -0.058 | -0.064 | -0.058 |  |
|  | (0.054) | (0.055) | (0.054) | (0.054) | (0.053) | (0.054) |  |
| Other race/ethnicity | 0.100+ | 0.098+ | 0.099+ | 0.098+ | 0.099+ | 0.096+ |  |
|  | (0.058) | (0.057) | (0.058) | (0.057) | (0.057) | (0.057) |  |
| Resides with two biological parents | -0.033 | -0.033 | -0.033 | -0.033 | -0.033 | -0.033 |  |
|  | (0.022) | (0.022) | (0.022) | (0.022) | (0.022) | (0.022) |  |
| Family socioeconomic status | 0.011 | 0.012 | 0.011 | 0.012 | 0.012 | 0.012 |  |
|  | (0.012) | (0.012) | (0.012) | (0.012) | (0.012) | (0.012) |  |
| Parent relationship quality | -0.042** | -0.042** | -0.042** | -0.043** | -0.042** | -0.042** |  |
|  | (0.013) | (0.013) | (0.013) | (0.013) | (0.013) | (0.013) |  |
| Neighborhood monitoring | -0.009 | -0.009 | -0.009 | -0.009 | -0.009 | -0.009 |  |
|  | (0.012) | (0.012) | (0.012) | (0.012) | (0.012) | (0.012) |  |
| Impulsivity | 0.002 | 0.001 | 0.002 | 0.001 | 0.001 | 0.001 |  |
|  | (0.013) | (0.013) | (0.013) | (0.013) | (0.013) | (0.013) |  |
| GPA | -0.048*** | -0.048*** | -0.048*** | -0.047*** | -0.048*** | -0.048*** |  |
|  | (0.012) | (0.012) | (0.012) | (0.012) | (0.012) | (0.012) |  |
| Suspended | 0.174*** | 0.174*** | 0.173*** | 0.174*** | 0.172*** | 0.171*** |  |
|  | (0.026) | (0.026) | (0.026) | (0.026) | (0.026) | (0.026) |  |
| Personal network size | 0.001 | 0.001 | 0.001 | 0.001 | 0.001 | 0.001 |  |
|  | (0.003) | (0.003) | (0.003) | (0.003) | (0.003) | (0.003) |  |
| No network data | 0.047 | 0.021 | 0.046 | 0.024 | 0.046 | 0.035 |  |
|  | (0.035) | (0.032) | (0.035) | (0.033) | (0.034) | (0.032) |  |
| Neighborhood socioeconomic disadvantage | 0.019 | 0.019 | 0.022 | 0.022 | 0.017 | 0.018 |  |
|  | (0.017) | (0.017) | (0.018) | (0.018) | (0.018) | (0.018) |  |
| Neighborhood residential instability | 0.007 | 0.008 | 0.006 | 0.007 | 0.007 | 0.007 |  |
|  | (0.016) | (0.017) | (0.016) | (0.017) | (0.017) | (0.017) |  |
| School-level Measures |  |  |  |  |  |  |  |
| County Population density | 0.002 | 0.002 | 0.001 | 0.000 | 0.004 | 0.004 |  |
|  | (0.007) | (0.007) | (0.007) | (0.007) | (0.007) | (0.007) |  |
| School socioeconomic disadvantage | -0.029 | -0.029 | -0.031 | -0.030 | -0.029 | -0.028 |  |
|  | (0.025) | (0.025) | (0.025) | (0.025) | (0.024) | (0.024) |  |
| %Black | 0.005 | 0.006 | 0.005 | 0.005 | 0.004 | 0.004 |  |
|  | (0.018) | (0.018) | (0.019) | (0.018) | (0.018) | (0.018) |  |
| %Hispanic | 0.063* | 0.063* | 0.063* | 0.063* | 0.062* | 0.062* |  |
|  | (0.029) | (0.028) | (0.029) | (0.029) | (0.029) | (0.029) |  |
| Pupil-teacher ratio | 0.001 | 0.001 | 0.001 | 0.001 | 0.001 | 0.001 |  |
|  | (0.004) | (0.004) | (0.004) | (0.004) | (0.004) | (0.004) |  |
| School suspension rate | 0.157 | 0.165 | 0.075 | 0.079 | 0.205 | 0.203 |  |
|  | (0.118) | (0.120) | (0.112) | (0.114) | (0.130) | (0.130) |  |
| Student population size | -0.000 | -0.000 | -0.000 | -0.000 | -0.000 | -0.000 |  |
|  | (0.000) | (0.000) | (0.000) | (0.000) | (0.000) | (0.000) |  |
| Private school | -0.063 | -0.072 | -0.055 | -0.059 | -0.067 | -0.074 |  |
|  | (0.052) | (0.053) | (0.050) | (0.051) | (0.053) | (0.054) |  |
| School-level Social Processes |  |  |  |  |  |  |  |
| Positive school climate | 0.018 | 0.015 |  |  |  |  |  |
|  | (0.020) | (0.015) |  |  |  |  |  |
| Relative network density | 0.005 | 0.005 | 0.008 | 0.009 | 0.001 | 0.004 |  |
|  | (0.017) | (0.018) | (0.016) | (0.017) | (0.019) | (0.019) |  |
| Low interpersonal trouble |  |  | 0.001 | -0.008 |  |  |  |
|  |  |  | (0.023) | (0.018) |  |  |  |
| School community attachment |  |  |  |  | 0.019 | 0.025 |  |
|  |  |  |  |  | (0.018) | (0.016) |  |
| Interactions |  |  |  |  |  |  |  |
| Positive school climate * Network density | 0.021* | 0.009 |  |  |  |  |  |
|  | (0.009) | (0.008) |  |  |  |  |  |
| Low interpersonal trouble * Network density |  |  | 0.026* | 0.012 |  |  |  |
|  |  |  | (0.011) | (0.011) |  |  |  |
| School community attachment * Network density |  |  |  |  | 0.011 | 0.001 |  |
|  |  |  |  |  | (0.011) | (0.011) |  |
| Personal network * Positive school climate | -0.002 |  |  |  |  |  |  |
|  | (0.002) |  |  |  |  |  |  |
| Personal network * Low interpersonal trouble |  |  | -0.002 |  |  |  |  |
|  |  |  | (0.002) |  |  |  |  |
| Personal network * School community attachment |  |  |  |  | -0.000 |  |  |
|  |  |  |  |  | (0.002) |  |  |
| No network data * Positive school climate |  | -0.014 |  |  |  |  |  |
|  |  | (0.022) |  |  |  |  |  |
| No network data * Network density |  | 0.015 |  | 0.007 |  | 0.002 |  |
|  |  | (0.029) |  | (0.028) |  | (0.028) |  |
| No network data * Positive school climate * Network density |  | 0.038* |  |  |  |  |  |
|  |  | (0.018) |  |  |  |  |  |
| No network data * Low interpersonal trouble |  |  |  | 0.011 |  |  |  |
|  |  |  |  | (0.022) |  |  |  |
| No network data * Low interpersonal trouble * Network density |  |  |  | 0.053* |  |  |  |
|  |  |  |  | (0.021) |  |  |  |
| No network data * School community attachment |  |  |  |  |  | -0.031 |  |
|  |  |  |  |  |  | (0.022) |  |
| No network data * School community attachment * Network density |  |  |  |  |  | 0.028 |  |
|  |  |  |  |  |  | (0.020) |  |
| Added Individual-level Variables |  |  |  |  |  |  |  |
| Wave I violence | 0.447*** | 0.447*** | 0.447*** | 0.446*** | 0.448*** | 0.447*** |  |
|  | (0.014) | (0.014) | (0.014) | (0.014) | (0.014) | (0.014) |  |
| Interpersonal trouble with students | 0.023+ | 0.023+ | 0.022+ | 0.023+ | 0.022+ | 0.022+ |  |
|  | (0.013) | (0.013) | (0.013) | (0.013) | (0.013) | (0.013) |  |
| Interpersonal trouble with teachers | 0.028* | 0.028* | 0.028* | 0.029* | 0.028* | 0.028* |  |
|  | (0.012) | (0.012) | (0.012) | (0.012) | (0.012) | (0.012) |  |
| School attachment | 0.028+ | 0.028+ | 0.029+ | 0.029* | 0.027+ | 0.028+ |  |
|  | (0.015) | (0.015) | (0.015) | (0.015) | (0.015) | (0.015) |  |
| Teacher fairness | -0.025+ | -0.026+ | -0.025+ | -0.025+ | -0.026* | -0.027* |  |
|  | (0.013) | (0.013) | (0.013) | (0.013) | (0.013) | (0.013) |  |
| (Intercept) | 0.553 | 0.634 | 0.453 | 0.550 | 0.356 | 0.327 |  |
|  | (0.765) | (0.760) | (0.781) | (0.778) | (0.769) | (0.770) |  |
| N (Respondents) | 11771 | 11771 | 11771 | 11771 | 11771 | 11771 |  |
| N (Schools) | 113 | 113 | 113 | 113 | 113 | 113 |  |
| *** p < 0.001; ** p < 0.01; * p < 0.05; + p < 0.1. | | | |  |  |  |  |
| Notes: Coefficients with errors in parentheses. All continuous independent variables are z-score standardized (mean = 0, SD = 1) except for personal network size, county population density, pupil-teacher ratio, personal network size, age, and school student population. Models are weighted according to Add Health Guidelines using the Stata "svyset" command. | | | | | | |  |
|  |  |  |  |  |  |  |  |
|  |  |  |  |  |  |  |  |

Appendix Figure 8. Respondent Network Interaction Models: Average Marginal Effects (AME) of Positive School Climate and Relative Network Density at Percentiles of One Another.


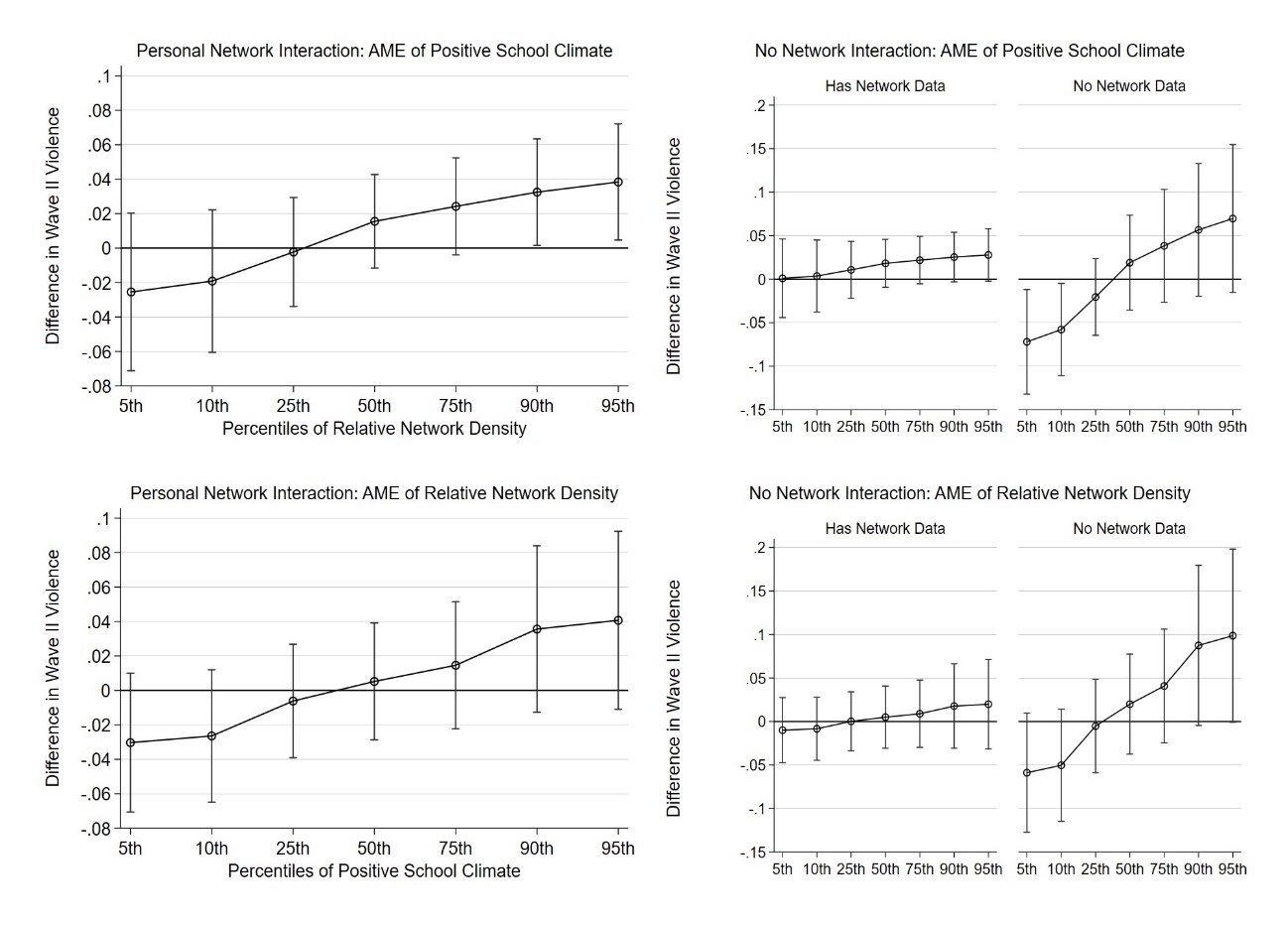


Appendix Figure 9. Respondent Network Interaction Models: Average Marginal Effects (AME) of Low Interpersonal Trouble and Relative Network Density at Percentiles of One Another.


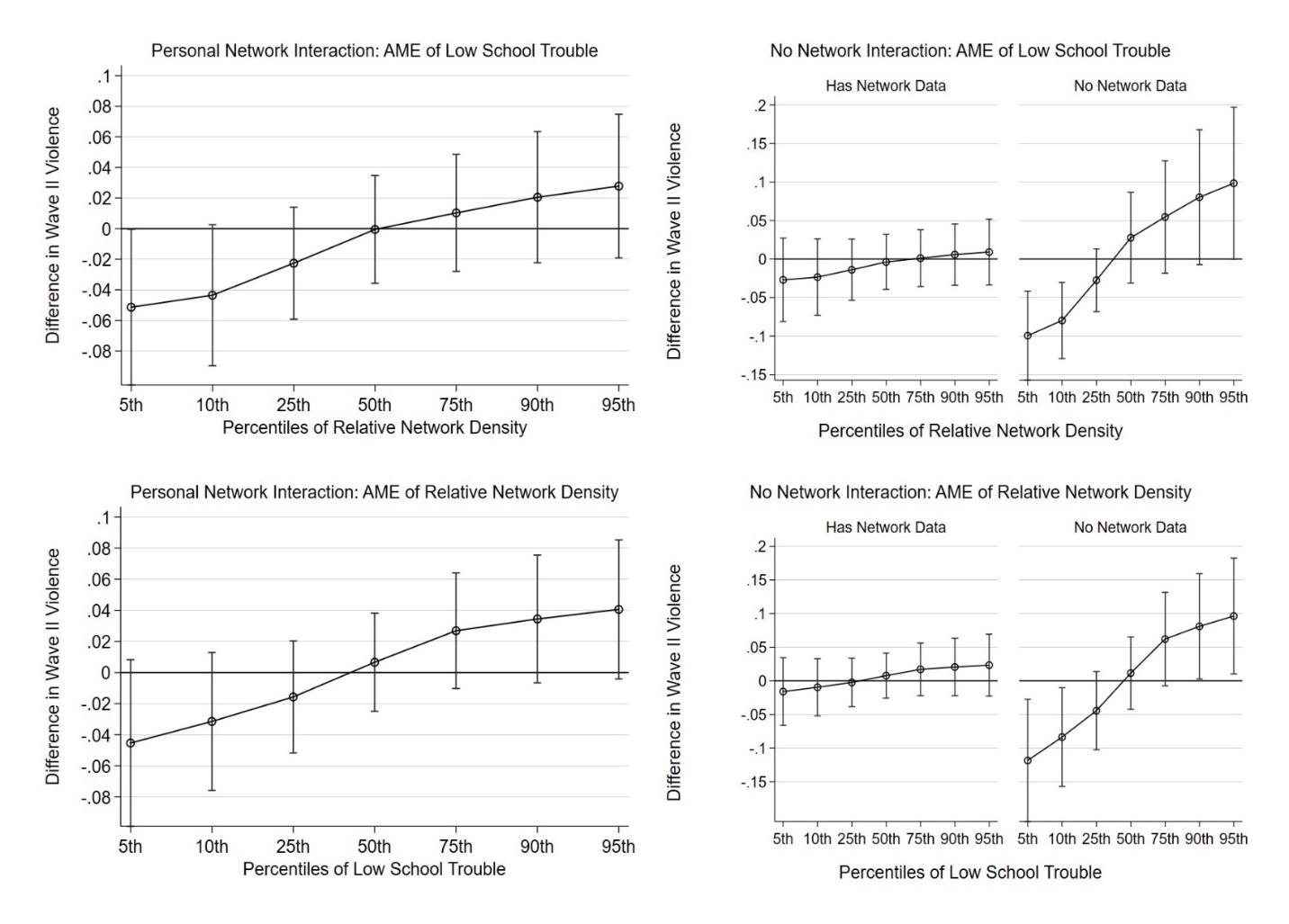


Appendix Figure 10. Respondent Network Interaction Models: Average Marginal Effects (AME) of School Community Attachment and Relative Network Density at Percentiles of One Another.


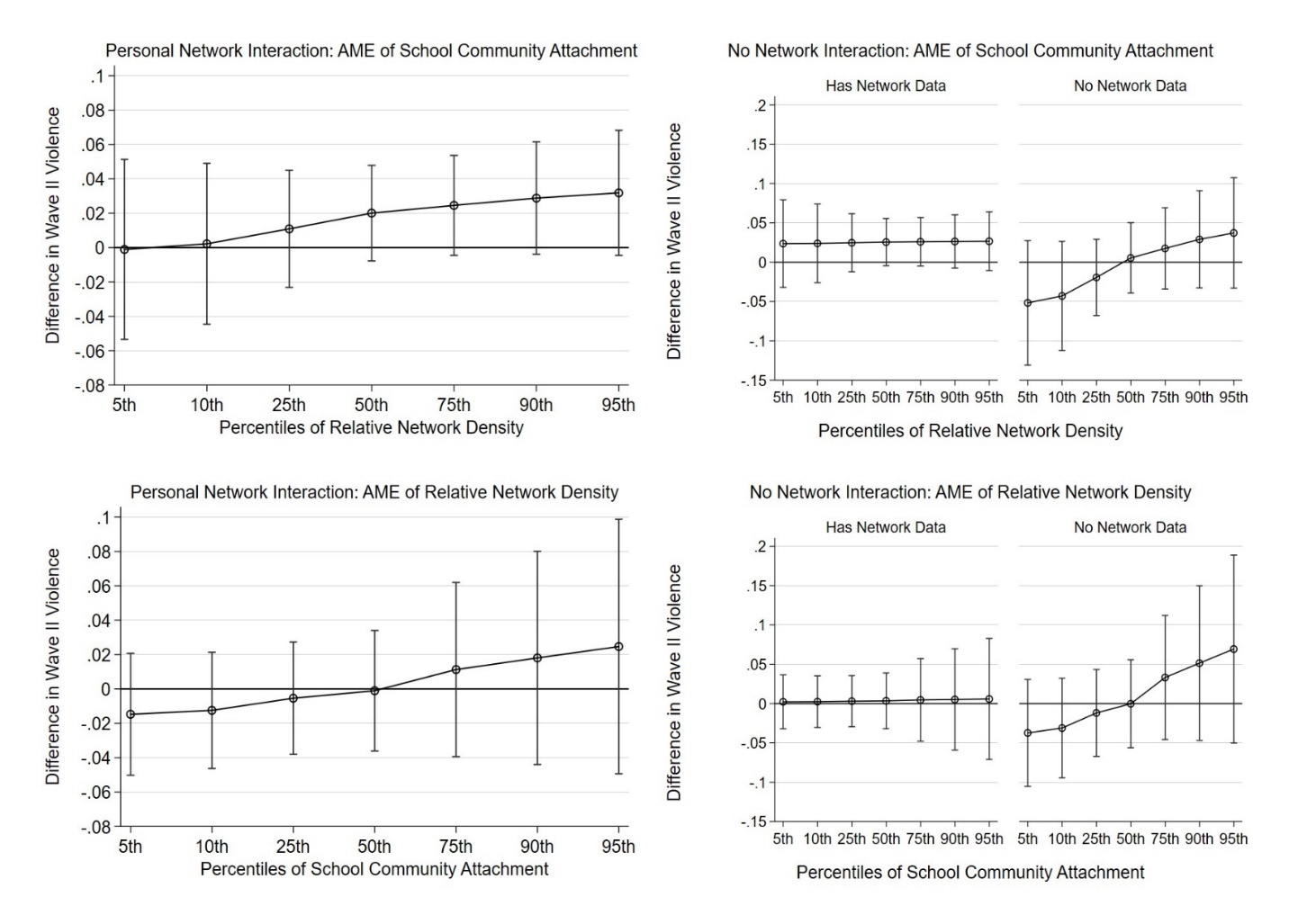

Supplement: Supplementary file 1 — Appendix [file 10964_2024_2034_MOESM1_ESM.docx]
